# Confined water-mediated high proton conduction in hydrophobic channel of a synthetic nanotube

**DOI:** 10.1038/s41467-020-14627-z

**Published:** 2020-02-18

**Authors:** Ken-ichi Otake, Kazuya Otsubo, Tokutaro Komatsu, Shun Dekura, Jared M. Taylor, Ryuichi Ikeda, Kunihisa Sugimoto, Akihiko Fujiwara, Chien-Pin Chou, Aditya Wibawa Sakti, Yoshifumi Nishimura, Hiromi Nakai, Hiroshi Kitagawa

**Affiliations:** 1 Division of Chemistry, Graduate School of Science, Kyoto University, Kitashirakawa-Oiwakecho, Sakyo-ku, Kyoto 606-8502 Japan; 2 Japan Synchrotron Radiation Research Institute (JASRI), SPring-8, 1-1-1 Kouto, Sayo-cho, Sayo-gun, Hyogo 679-5198 Japan; 3 Research Institute for Science and Engineering, Waseda University, Shinjuku-ku Tokyo, 169-8555 Japan; 4 Department of Chemistry and Biochemistry, School of Advance Science and Engineering, Waseda University, Shinjuku-ku Tokyo, 169-8555 Japan; 5 Element Strategy Initiative for Catalysts and Batteries (ESICB), Kyotodaigaku-Katsura, Kyoto University, Kyoto, 615-8520 Japan; 6 Present Address: Institute for Integrated Cell-Material Sciences, Kyoto University, Yoshida, Sakyo-ku Kyoto, 606-8501 Japan; 7 Present Address: Institute of Liberal Education, School of Medicine, Nihon University, Oyaguchi Uemachi 30-1, Itabashi-ku Tokyo, 173-8610 Japan; 8 Present Address: The Institute for Solid State Physics, The University of Tokyo, Kashiwa, Japan; 9 Present Address: Department of Chemistry, University of Calgary, Calgary, Alberta T2N 1N4 Canada; 10 Department of Nanotechnology for Sustainable Energy School of Science and Technology, Kwansei Gakuin University, 2-1 Gakuen, Sanda Hyogo, 669-1337 Japan

**Keywords:** Reaction kinetics and dynamics, Nanoscale materials

## Abstract

Water confined within one-dimensional (1D) hydrophobic nanochannels has attracted significant interest due to its unusual structure and dynamic properties. As a representative system, water-filled carbon nanotubes (CNTs) are generally studied, but direct observation of the crystal structure and proton transport is difficult for CNTs due to their poor crystallinity and high electron conduction. Here, we report the direct observation of a unique water-cluster structure and high proton conduction realized in a metal-organic nanotube, [Pt(dach)(bpy)Br]_4_(SO_4_)_4_·32H_2_O (dach: (1R, 2R)-(–)-1,2-diaminocyclohexane; bpy: 4,4’-bipyridine). In the crystalline state, a hydrogen-bonded ice nanotube composed of water tetramers and octamers is found within the hydrophobic nanochannel. Single-crystal impedance measurements along the channel direction reveal a high proton conduction of 10^−2^ Scm^−1^. Moreover, fast proton diffusion and continuous liquid-to-solid transition are confirmed using solid-state ^1^H-NMR measurements. Our study provides valuable insight into the structural and dynamical properties of confined water within 1D hydrophobic nanochannels.

## Abstract (web summary)

Water confined in natural or synthetic hydrophobic nano-spaces behaves differently than in the bulk. Here the authors investigate water in hydrophobic synthetic 1D nanochannels revealing water clustering in tetramers and octamers and high proton conductivity, along with a continuous liquid to solid transition.

## Introduction

Water confined within a hydrophobic nanospace has been of particular interest for researchers due to the unusual structural and dynamical properties, which differ significantly from those of bulk water^1,2^. In a hydrophobic nanochannel with a small aperture size (<3−4 nm), confined water shows remarkable physical properties which arise from the reduced number of neighbouring water molecules, resulting in a restricted hydrogen-bond network^2,3^. To study the effect of confining water in a one-dimensional (1D) nanospace, water-filled carbon nanotubes (CNTs) have been an attractive research focus, with interesting water-cluster formation^4,5^, fast water transport^6,7^, and high proton conductivity observed^8–10^. Such studies on the dynamics of water confinement in hydrophobic nanochannels are especially important as biomimetics^11,12^ for understanding transport mechanisms in proton pumps^13^ or water transport proteins^14^. However, the poor crystallinity and high electrical conductivity of CNTs cause difficulties for detailed investigation of the structure and proton transport properties of confined water within the 1D hydrophobic nanochannels.

In contrast to CNTs, structural fabrication of nanochannels using coordination chemistry is a promising approach due to the richness in structural degrees of freedom and the relative ease of structural characterisation through crystallographic techniques. Among coordination complexes, metal-organic frameworks (MOFs), which are infinite coordination networks of metal ions bridged through organic ligands, have attracted much interest due to their structural designability and uniform porosity^15–17^. Recently, we have developed a rational bottom-up synthesis of a metal-organic nanotube^18^ based on the oxidative polymerisation of a square-shaped platinum complex using elemental iodine. This nanotube is composed of four 1D halogen-bridged transition-metal chains (MX-chains) which are connected linearly bridging organic ligands to form a right square prism-shaped nanotubular structure (MX-tube)^19^.

**Fig. 1:**
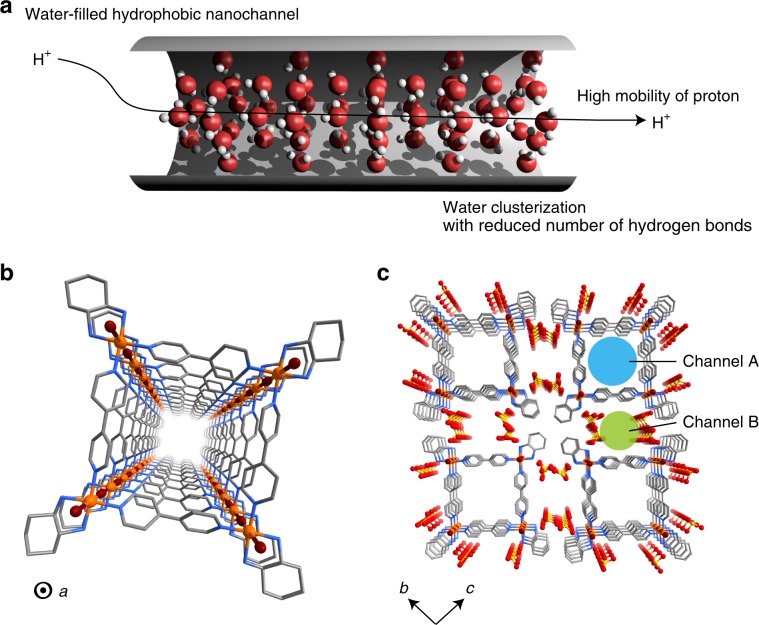
Proton-conducting hydrophobic nanochannel. **a** Schematic illustration of a water-filled hydrophobic nanochannel. **b** The four-legged tubular structure of **1** viewed along the *a*-axis (100 K). Counter anions and water molecules have been omitted for clarity. **c** The packing structure of **1** in the *bc* plane. Channel A and channel B are highlighted by light blue and light green circles, respectively. Water molecules are omitted for clarity. Platinum, bromine, sulfur, carbon and nitrogen are represented by orange, brown, yellow, grey and blue spheres, respectively.

Herein, we present the direct observation of the proton dynamics of water confined in the hydrophobic channel of a synthetic nanotubular material^20,21^ (Fig. 1a). We synthesise the metal-organic nanotube [Pt(dach)(bpy)Br]_4_(SO_4_)_4_·32H_2_O (**1**, dach: (1*R*, 2*R*)-(-)-1,2-diaminocyclohexane; bpy: 4,4′-bipyridine), and elucidate unique water clustering composed of water tetramers and octamers within the hydrophobic nanochannel of **1** using single-crystal X-ray analyses. Single-crystal impedance spectroscopy measurements and solid-state ^1^H-NMR studies, both supported by theoretical calculations, are used to show high proton conductivity of **1** that originates from the confined water within the hydrophobic nanochannel. In addition, we find a continuous liquid-to-solid transition of the confined water, which is significantly different from that of bulk water.

## Results

### Confined water in the nanochannel of a metal-organic nanotube

**Fig. 2:**
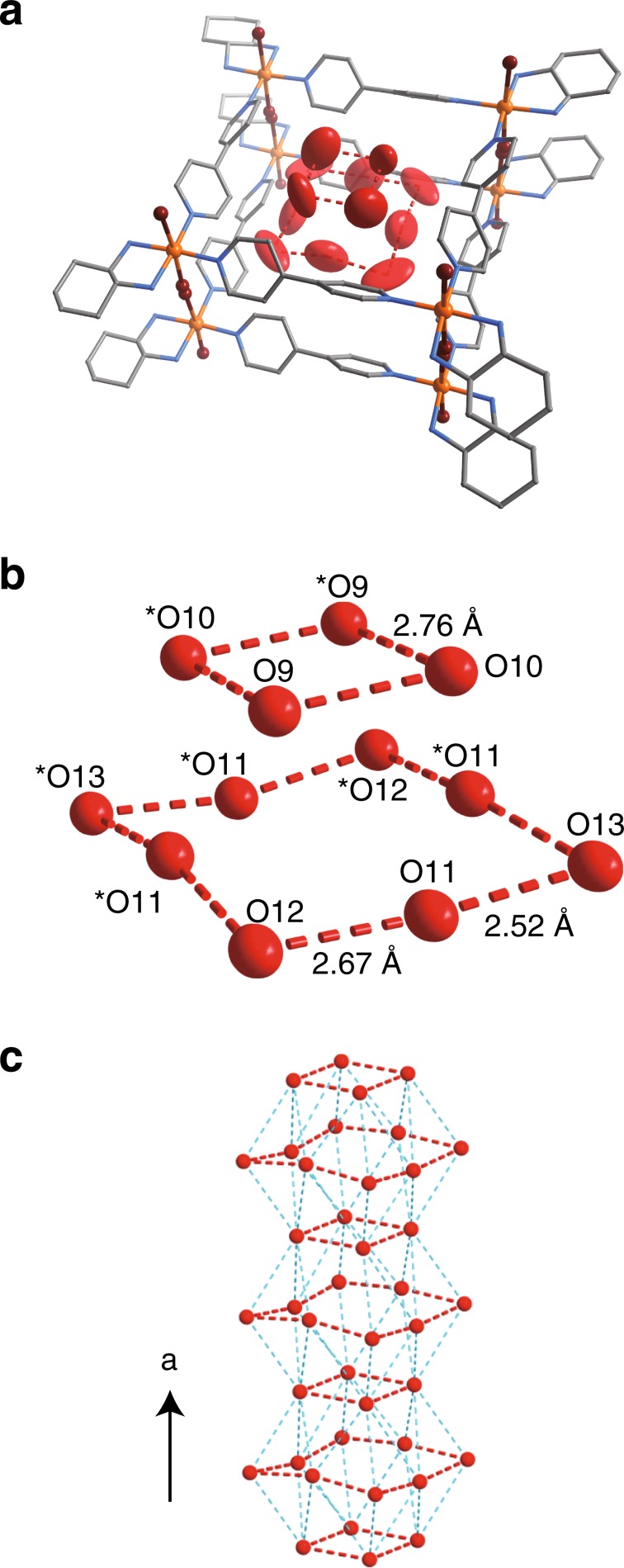
Water-cluster formation within channel A (100 K). The results of SCXRD are displayed. **a** ORTEP drawing (30% probability ellipsoids) of the 12 unique oxygen atoms in the nanotube. **b** Tetramer and octamer-like water clusters. The red dotted line represents the hydrogen bonds. **c** 1D hydrogen-bonding network of water clusters. Light blue dotted line represents the hydrogen bonds between water clusters.

Single crystals of **1** were obtained by the oxidative polymerisation of a square-shaped complex, [Pt(dach)(bpy)]_4_(SO_4_)_4_, using elemental Br_2_ (Supplementary Figs. 1–7, see Supplementary Information for details). The crystal structure of **1** was determined by single-crystal X-ray diffraction (SCXRD) at 100 K (Fig. 1b, c, Supplementary Figs. 8–16, and Supplementary Table 1). The structure consists of symmetrically equivalent nanotubes, with square [Pt(dach)(bpy)]_4_ units bridged to each other along through bromide anions at the corners of the square, resulting in tubes that propagate parallel to one another along the *a-*axis. Overall, there is a net 8+ charge per square [Pt(dach)(bpy)Br]_4_ unit, which is charge-balanced by four sulfate anions that reside between the tubes. The tubes are spaced 14.1 Å (17.0 Å) apart along the *b*- and *c*-direction and are propped apart due to steric interactions between the bulky exterior dach ligands and sulfate counter anions. This spacing leaves two types of water-filled 1D channels in the crystal, a hydrophobic interior channel A and a hydrophilic exterior channel B (Fig. 1c). Within channel B, water molecules form a 1D hydrogen-bond network which includes terminal dach amino groups and sulfate anions (Supplementary Fig. 9). Within channel A on the other hand, water forms two unique clusters of alternating octamers and tetramers, having strong hydrogen-bond interactions within the cluster and weaker interactions between clusters (Fig. 2). These tetramers and octamers are similar to theoretically-predicted conformations of small water clusters^22^. There are three crystallographically-unique water molecules in the octamer and two in the tetramer, and medium strength hydrogen bonds hold each cluster together at 2.5–2.7 Å O–O distances. The clusters form weak hydrogen bonds between one another in the channel direction at distances of 3.0–3.4 Å. Water in the clusters are separated from the walls of the tube by >2.9 Å, indicating weak contact forces between the clusters and the inner surface of the nanochannel. This clustering arises from the hydrophobic nature of the channel, which is similar to that of CNTs or biological channels in ion transport proteins^6,13,14^. In **1**, the alternating M-X nature of the tube gives rise to a corrugated interior which narrows near the bpy surfaces, which is in contrast to the smooth interior surface of a CNT (Supplementary Fig. 12). Variable-temperature SCXRD was used to understand the dynamics of the water clusters with increasing temperature; water-clusters were clearly observed below 200 K, but became slightly disordered above 250 K (Supplementary Fig. 13).

### High proton conductivity

**Fig. 3:**
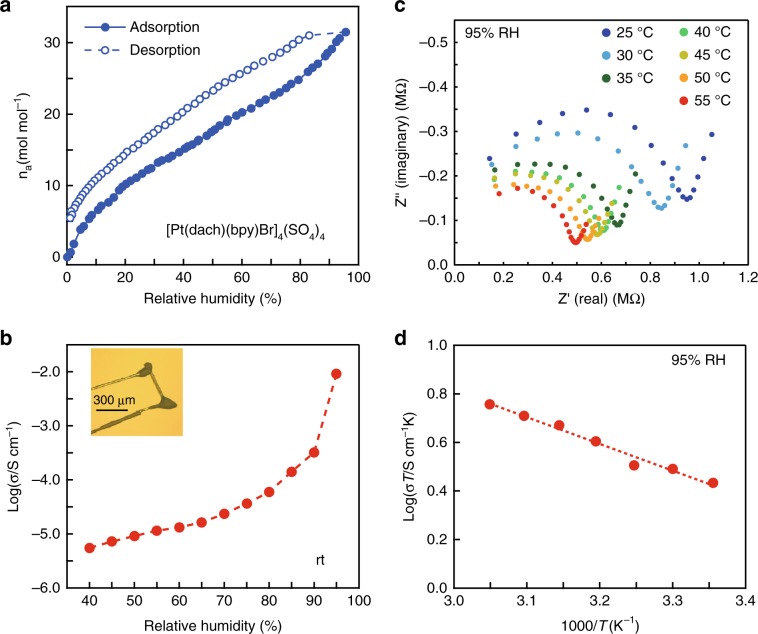
Humidity dependence of proton conductivity of **1.** **a** Water vapour sorption isotherms (298 K). The blue open and closed circles denote adsorption and desorption processes, respectively. **b** Humidity dependence of conductivity: Inset shows the experimental setup. **c** Nyquist plots of **1** at elevated temperature under a relative humidity of 95%. **d** Arrhenius plots of the conductivity under a relative humidity of 95%.

To investigate any unique transport properties arising from the nanoconfinement of water, impedance spectroscopy measurements on a single crystal of **1** were performed along the channel direction (*a*-direction; Fig. 3b inset). We first confirmed that the dc conductivity is less than the lower limit of measurement, ruling out significant electrical conductivity in the tube. On the other hand, when measured with ac impedance spectroscopy, **1** gave a semi-circular response in the Nyquist plot, suggesting that the conductivity is protonic in nature. Upon increasing the relative humidity (RH) from 40 to 95%, the conductivity increased significantly by over three orders of magnitude (Fig. 3b). This strong humidity dependence clearly suggests that the included water molecules are essential for the high level of conductivity. At 55 °C and 95% RH, the conductivity of **1** reached 1.7 × 10^−2^ S cm^−1^ (Fig. 3c). This value is quite high and in the range of superprotonic conductivity, which is comparable to the commercial proton-exchange polymer membrane Nafion^23^. This high level of conductivity means that **1** is among the most proton-conductive MOFs despite the fact that **1** does not have any free functional acidic groups or free acids inside the pores.^24,25^ The proton conductivity measured on a pelletised powder sample (Supplementary Figs. 17–20) was about a hundredth lower than that measured with a single crystal at 95% RH, suggesting that the proton conduction is highly anisotropic and that the conducting pathway is in the channel direction. The conductivity showed Arrhenius behaviour with varying temperature, with an activation energy (*E*_a_) of 0.22 eV (Fig. 3d). This low *E*_*a*_ is close to those of typical proton-conductive MOFs showing Grotthuss-type proton hopping, where a protonic charge defect diffuses through the hydrogen-bond network^17,26,27^. Moreover, the water-cluster structures of **1** were retained above 200 K (~250 K, Supplementary Fig. 13), indicating that high proton conductivity is derived not from mobile hydronium ions (H_3_O^+^) themselves but rather from proton shuttling through the hydrogen-bond network between interacting water clusters.

### Proton-conducting mechanism

**Fig. 4:**
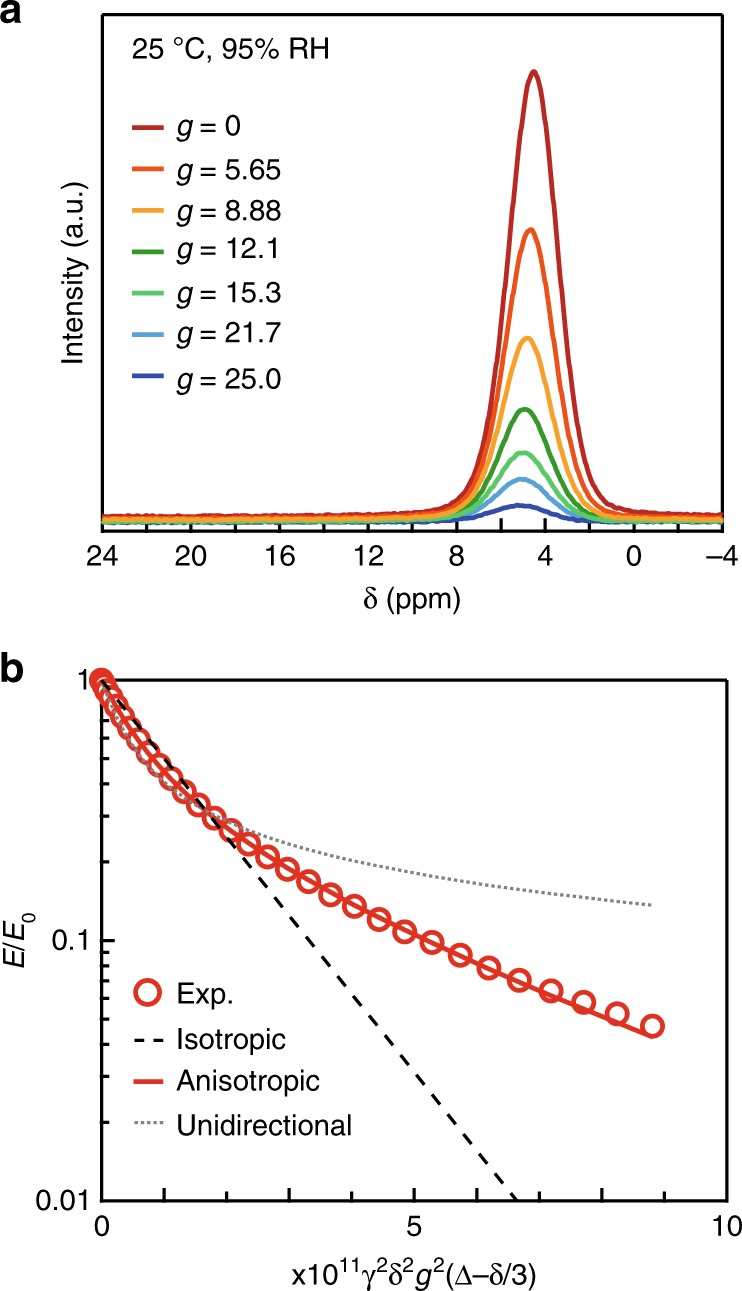
Intensity decay of the echo of the crystallization water of **1**. **a** Echo decay with selected *g* values at 25 °C, 95% RH. **b** Fit to the attenuation of the normalized signal intensity (*E*/*E*_0_) as a function of *g*^2^ based on the two-component anisotropic diffusion model (see text). Here, the gradient pulse duration (δ) is 1 ms, and the diffusion time (Δ) is 20 ms. γ is the gyromagnetic ratio of ^1^H. Grey dotted line, red solid line and black dashed line are the best fits to the experimental data (red circle) using isotropic, anisotropic and unidirectional diffusion model, respectively.

For further investigation of the transport mechanism of **1**, solid-state ^1^H-NMR measurements were performed (Supplementary Figs. 21–27). In the ^1^H-MAS NMR spectra, the peak of the crystallization water was observed as a single component, indicating the rapid exchange of protons of water between channel A and B (Supplementary Fig. 22). The self-diffusion coefficient of ^1^H in a water molecule can be estimated by the pulsed-field gradient (PFG) method^28–30^, by which the diffusion movement is detected as the attenuation of an observed echo signal. Because the self-diffusion coefficient of ^1^H in water has a linear relationship with the mobility of the proton carrier in proton conduction, PFG-NMR measurements provide useful insight into the mechanism of conduction. Figure 4 shows the results of PFG-NMR measurements at 25 °C and 95% RH. The peak centred at 4.54 ppm was assigned as crystallization water protons and was observed to decay with the increasing strength of the gradient pulse (*g*) (Fig. 4a and Supplementary Figs. 23–27). The echo attenuation follows different equations depending on the dimensionality of the diffusion path (isotropic, anisotropic, or unidirectional; see Supplementary Materials for detail)^29–31^, and in our case, the curve fitting was successful when considering the single component of an anisotropic diffusion model (Fig. 4b) expressed as,

$$E/E_0 = \exp \left( { - kD_ \bot } \right)\int_0^1 {{\mathrm{exp}}} ( - k[D_{||} - D_ \bot ]x^2){\mathrm{d}}x$$

where *k* = γ^2^δ^2^*γ*^2^(Δ−δ/3), and *D* is the self-diffusion coefficient. The subscripts of *D* represent the diffusion parallel (*D*_||_) and perpendicular (*D*_⊥_) to the channel alignment direction. The fitting result gave self-diffusion coefficients of *D*_||_ = 2.9 × 10^−11^ m^2^s^−1^ and *D*_⊥_ = 1.6 × 10^−12^ m^2^s^−1^. The diffusion time (Δ) dependence of *D* and mean-square displacement of ^1^H also supports the anisotropic nature of the diffusion (Supplementary Figs. 24 and 25). The self-diffusion coefficient of *D*_||_ is comparable to that reported for Nafion (0.2–20 × 10^–10^ m^2^s^–1^; *D* of Nafion largely varies with water mass fraction)^32^. From the temperature dependence of self-diffusion coefficients, the activation energies are estimated to be 0.18 eV for *D*_||_ and 0.14 eV for *D*_⊥_ (Supplementary Fig. 26). Compared to the impedance result, smaller activation energies were likely determined because PFG-NMR observes shorter-range diffusion. It should be noted that the observed *D*_||_ contains the contributions from both channel A and B. The very smooth proton diffusivity in the hydrophobic nanochannel A is expected because of weak interactions among the confined waters and the inner wall of the tube^6–10^, resulting in the lowering of the potential barrier heights of the rotational and translational movements of the included water molecules. Conversely, moderate diffusivity in channel B would be expected due to the low dimensionality of the diffusion path and the large number of hydrogen bonds among the water molecules, sulfate anions, and amines of the tubular framework, which restricts water reorientation and diffusion^29^. In addition, our recent study on related nanotubular analogues having ultrasmall diameters revealed that the observed proton conductivities are much lower than that of **1**^33^. In these analogue compounds, no water molecule exists inside the hydrophobic nanotube, whereas the hydrophilic channel outside of the nanotube contains several water molecules. These results suggest that the existence of confined waters within hydrophobic channel A with smooth proton diffusivity plays an important role in the realization of the high proton conductivity of **1**.

As the conductivity (*σ*) can be expressed as *σ* = *neμ* (*n*: the number of carriers, *e*: charge, *μ*: mobility of carriers), the existence of mobile proton sources is very important for attaining high proton conduction. In our case, the amine sites of the dach ligand that are coordinated to the Pt^IV^ sites could be considered as possible proton sources. Compared to the p*K*_a_ value of free amines (p*K*_a_ = 25–30), the p*K*_a_ of amines coordinated to metal ions are known to be much lower^34^. DFT calculations clearly support this assumption and indicate that the amine groups coordinated to Pt^IV^ cations in **1** have similar acidity to carbonic acid (Supplementary Figs. 28 and 29 and Supplementary Table 3).

To obtain atomistic insights on the proton diffusivity in the hydrophobic nanochannel, we performed quantum-mechanical molecular dynamics (QM-MD) simulations (Supplementary Figs. 30–35, Supplementary Tables 4–6 and Supplementary Movies 1–3; see Supplementary Materials for details). The vehicular diffusion^35^ coefficients (*D*_v_) estimated from the time dependence of mean square displacement indicate that water molecules in channel A move approximately 1.5 times faster than those in channel B (Supplementary Fig. 35a). According to the decomposition into parallel and perpendicular contributions with respect to channel alignment direction, the vehicular diffusion is not completely isotropic in both channel A and B (Supplementary Table 7). The Grotthuss diffusion^26,27^ coefficients (*D*_G_) were calculated based on the proton transfer rate, which was quantified by counting proton shuttling at each simulation step^36,37^. The proton transfer rate in channel A was more than two times higher that of channel B because attractive interaction with the sulfate anion in channel B hindered the proton shuttling (Supplementary Fig. 35b). The averaged value of *D*_G_ obtained from channel A and B accounts for the larger portion in overall proton diffusion coefficient (*D*_p_) given by the summation of *D*_G_ and *D*_v_^38–40^. The comparison of *D*_p_ among three water environments showed that the proton diffusivity in the hydrophobic nanochannel is in between the liquid and solid state (Supplementary Table 8).

### Liquid-to-solid phase transition of confined water

**Fig. 5:**
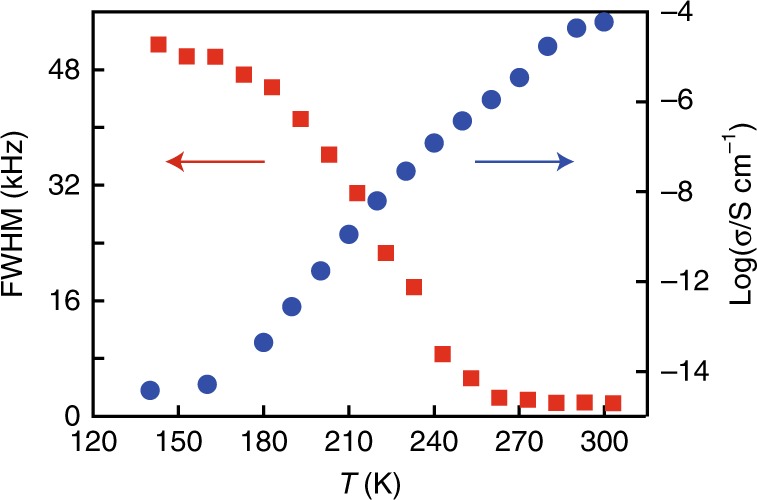
Temperature dependence of ^1^H NMR spectra and proton conductivity. Red squares and blue circles indicate FWHM of ^1^H NMR spectra (Supplementary Fig. 37) and proton conductivity (pelletised sample, Supplementary Fig. 38), respectively.

The liquid-to-solid phase transition behaviour of confined water has been of interest because the nature of the freezing of water in quasi-one-dimensional systems is not simple and has not been fully understood^4,5,41,42^. The experimental and theoretical investigations of CNTs have indicated that the phase transition behaviour strongly depends on the pore diameter and pressure; the transition behaviour has been predicted to be discontinuous (first-order-like) for a pore diameter of >1.2 nm and continuous for a pore diameter of <1.2 nm at ambient pressure^43^. We investigated the liquid-to-solid phase transition behaviour of confined water in **1** using differential scanning calorimetry (DSC), solid-state ^1^H-NMR and impedance measurements at low temperature (Supplementary Figs. 36–39). The DSC measurements showed no obvious phase transition (ice transition or melting) of the confined water between 163 and 293 K (Supplementary Fig. 36), suggesting that the confined water shows a continuous liquid-to-solid transition in the channels. The temperature dependence of the solid-state ^1^H-NMR signal line width for the crystallisation water of **1** showed a gradual and continuous broadening as the temperature decreased (Supplementary Figs. 37 and 39). These results indicate that the confined water in **1** exhibits no clear liquid-to-solid phase transition, which is markedly different from the behaviour of the bulk water or water confined in micropores^5,43^. This is also supported by the proton conductivity showing a gradual increase with temperature, rather than an inflection indicative of a phase transition (Supplementary Fig. 38). From the combination of solid-state ^1^H-NMR and proton conductivity, the correlation between the proton conductivity and the continuous liquid-to-solid phase transition is clearly visualized (Fig. 5).

## Discussion

We have synthesised and characterised a metal-organic nanotube that has water-filled hydrophobic nanochannels in the crystalline state. The hydrophobic nanochannel of the nanotube has the square-shaped aperture with the size of *ca*. 1.0 nm (Fig. 1). The confined water in the hydrophobic nanochannel of **1** forms unique clustered structures, which was confirmed by SCXRD measurements. Single crystal impedance spectroscopy revealed that **1** exhibits very high proton conductivity along the 1D channel direction. The high conductivity of **1** is attributed to both the high proton diffusivity within the hydrophobic nanochannel combined with the acidity of the coordinated amine protons, both of which were supported by PFG-NMR and theoretical calculations. In addition, the continuous liquid-to-solid phase transition of the confined water in **1** was observed from DSC, solid-state ^1^H-NMR and impedance measurements, showing markedly different behaviour from bulk water. In 2001, Brewer et al.^8^, pointed out that the properties of confined water and proton transport through that water in a hydrophobic nanochannel are highly sensitive to the size of the channel. These properties of confined water originate from the reduced number of possible hydrogen-bond interaction in the nanochannel and negligible water–channel wall interaction^2,3^, which become similar to those of bulk water as the diameter of the nanochannel become larger. Recent experimental results using CNTs also validated these theoretical predictions.^2,5,44^ For example, Noy et al., experimentally demonstrated that 0.8-nm-diameter CNT porins show extraordinarily fast proton transport while 1.5-nm-diameter CNT porins show the proton transport rate comparable to bulk water^44^. Consistent with these previous works on CNTs with the aperture sizes of <1.5 nm, our nanotube experimentally demonstrated the unique clustering structures, high proton mobility, and a continuous liquid-to-solid phase transition of the confined water. The present results show strong experimental evidence of the predicted behaviour of confined water in hydrophobic nanochannels. Nanotube fabrication based on coordination chemistry can allow for systematic structural tuning of pore size, shape and surface properties. This tunability can thus enable us to systematically investigate the properties of confined water. We believe that these findings will provide valuable structural and dynamic insights about confined molecular species in nanotubular materials as well as in biological channels.

## Methods

### Syntheses and characterisation of compounds

Reagents and solvents were purchased from Wako Pure Chemical Industries, Ltd., TCI Co., Ltd., and Sigma-Aldrich Chemical Co. and used without further purification. Elemental analyses for all compounds were performed using Yanaco MT-5 and MT-6 CHN recorders at the Centre for Organic Elemental Microanalysis, Kyoto University.

### Synthesis of [Pt(dach)(bpy)Br]_4_(SO_4_)_4_·32H_2_O (1)

To an aqueous solution containing equimolar amounts of the square-shaped unit [Pt(dach)(bpy)]_4_^8+^ and its brominated square unit [Pt(dach)(bpy)Br_2_]_4_^8+^, an excess amount of tetrabutylammonium sulfate [(C_4_H_9_)_4_N]_2_(SO_4_) were added, and then the mixture was stirred at rt. A red-purple precipitate of **1** was collected by filtration (60% yield from the starting material (Pt(dach)(NO_3_)_2_)). Single crystals were obtained by slow tetrahydrofuran diffusion to an aqueous solution of the powder sample. Elemental analysis (%) calcd for C_64_H_88_N_16_O_16_S_4_Br_4_Pt_4_**·**28H_2_O: C 25.04, H 4.73, N 7.30, S 4.18; found: C 24.73, H 4.35, N 7.26 S 4.25. (IR spectrum and TGA curve are shown in Supplementary Figs. 4 and 5, respectively. See Supplementary Discussion for the experimental detail).

### Adsorption/desorption isotherms

The sorption experiments for H_2_O (298 K) were carried out using a BELSORP-max (MicrotracBEL). The as-synthesized sample **1** was dried under high vacuum (<10^−1^ Pa) at 35 °C for 2 days to remove the crystallisation water before measurements. Isotherms are shown in Fig. 2a.

### Single-crystal X-ray crystallography

An X-ray crystal structure analyses were carried out using a Bruker SMART APEX II CCD detector with graphite-monochromated Mo Kα radiation (*λ* = 0.71073 Å) at 100 K. Single crystals were prepared by the vapour diffusion method using a water solution of **1** and THF, as described above. Suitable single-crystals were selected in the mother liquid and quickly transferred to paraton oil to avoid undesired solvent loss. The single crystals were mounted on MicroMesh (MiTeGen). The structures were solved by direct methods (SIR92), expanded using Fourier techniques (DIRDIF99) and refined by full-matrix least-squares refinement on *F*^2^ (SHELXL-97) using the CrystalStructure software package. The refinement result for **1** is summarized in Supplementary Table 1.

### Impedance measurements

Impedance measurements were carried out with a Solartron SI 1260 Impedance/Gain-Phase Analyser and 1296 Dielectric Interface in the frequency range of 1–1 × 10^6^ Hz. The relative humidity and temperature were controlled by an Espec Corp. SH-221 incubator. The measurements in the low temperature region (140–300 K) were taken using Oxford OptistatDN2 and Lakeshore 340 temperature controllers.

### Solid-state ^1^H NMR measurements

Solid-state ^1^H nuclear magnetic resonance (NMR) measurements of **1** were performed on an AVANCE II^+^ 400 NMR spectrometer (Bruker Biospin K. K.) with an UltraShield^TM^ 400 WB 9.4 T superconducting magnet. ^1^H magic angle spinning (MAS) spectra and ^13^C cross-polarization (CP) MAS spectra were measured with a rotor of 4 mm diameter. Pulse field gradient (PFG)-NMR measurements were performed with a Diff 50 diffusion probe (Bruker Biospin K. K.).

## Supplementary information

Supplementary Information

Description of Additional Supplementary Files

Supplementary Data 1

Supplementary Data 2

Supplementary Data 3

Supplementary Movie 1

Supplementary Movie 2

Supplementary Movie 3

## Data Availability

The experiment data that support the findings of this study are available from the corresponding authors upon reasonable request. Crystallographic data in CIF format have been deposited in the Cambridge Crystallographic Data Centre (CCDC) under deposition numbers CCDC-1441696, 1441697, and 1441705. These data can be obtained free of charge via www.ccdc.cam.ac.uk/data_request/cif (or from the Cambridge Crystallographic Data Centre, 12 Union Road, Cambridge CB2 1EZ, U.K.).

## Code availability

The executable for running quantum-mechanical molecular dynamics simulations used in the present study can be downloaded for academic purpose via www.chem.waseda.ac.jp/dcdftbmd.
