# Supplementary material for: Confined water-mediated high proton conduction in hydrophobic channel of a synthetic nanotube
Source: Nat Commun. 2020 Feb 18;11:843. doi: 10.1038/s41467-020-14627-z (PMC7029035; doi:10.1038/s41467-020-14627-z)
Supplement: Supplementary file 1 — Supplementary Information [file 41467_2020_14627_MOESM1_ESM.pdf]

Supplementary Information

Confined Water-Mediated High Proton Conduction in Hydrophobic Channel of a Synthetic Nanotube

K. Otake *et al.*

Table of Contents

1. Materials and Methods **pp.3–60**

2-1. Syntheses

2-2. Crystal structure of 1

2-3. Electronic state of 1

2-4. Proton conductivity measurements

2-5. Solid-state ^{1}H NMR measurements

2-6. PFG NMR measurements

2-7. H^{+} dissociation energy

2-8. DFTB parameterization of Pt-N and Pt-Br for the nanochannel simulation

2-9. QM-MD simulation of confined water in hydrophobic nanochannel

2-10. Liquid-to-solid transition of confined water

3. References **pp.61–67**

1. Materials and Methods

Syntheses. Reagents and solvents were purchased from Wako Pure Chemical Industries, Ltd., TCI Co., Ltd., and Sigma-Aldrich Chemical Co., and used without further purification. Elemental analyses for all compounds were performing using Yanaco MT-5 and MT-6 CHN recorders at the Centre for Elementary Analysis, Kyoto University.

600 MHz ^{1}H-NMR measurements. ^{1}H-NMR spectra were recorded using a Bruker AVANCE-600 at room temperature. DMSO-d6 and D_2O were used as solvents.

Fourier transform infrared (FTIR) spectroscopy. FTIR spectra were recorded from a KBr pellets on a Thermo Fisher NEXUS 670 FTIR.

Thermogravimetric analysis. The thermogravimetric analysis of single crystals of **1** (ground in a mortar) was carried out using a TG-DTA 2000SA (Bruker AXS) at a heating rate of 3 K per min in a constant flow of N_2. Approximately 5 mg of sample was used for the measurement.

Adsorption/desorption isotherms. The sorption experiments for H_2O (298 K) and N_2 (77 K) were carried out using a BELSORP-max (MicrotracBEL). The as-synthesized sample **1** was dried under high vacuum ($<10^{-1}$ Pa) at 35 ˚C for 2 days to remove the crystallization water before measurements. Approximately 100 mg of sample was used for the measurement. Isotherms are shown in Figure 2 in the main pages.

Diffuse reflectance spectroscopy. Diffuse reflectance spectrum of **1** diluted in CaF_2 powder was recorded using a JASCO Model ISN-470 60 mmϕ integrating-sphere apparatus. The obtained reflectance spectra were converted to absorption spectra according to the Kubelka–Munk function $F(R_\infty)$.

Raman spectroscopy. The single-crystal Raman spectrum of **1** was recorded using a JASCO Model NRS-1000 with a microscope attachment. A Showa Optronics Model JUNO 532-100S He-Ne laser (632 nm) provided the exciting line, and each incident laser was polarized parallel to the MX-chain axis.

Single-crystal X-ray crystallography. X-ray crystal structure analysis was carried out using a Bruker SMART APEX II CCD detector with graphite-monochromated Mo Kα radiation (λ = 0.71073 Å) at 100 K. The single crystals were mounted on MicroMesh (MiTeGen) with Paratone oil. The structures were solved by direct methods (SIR92)[1], expanded using Fourier techniques (DIRDIF99)[2] and refined by full-matrix least-squares refinement on F^2 (SHELXL-97)[3] using the CrystalStructure software package[4]. The refinement result for **1** is summarized in Supplementary Table 1. Crystallographic data in CIF format have been deposited in the Cambridge Crystallographic Data Centre (CCDC) under deposition numbers CCDC-1441696, 1441697 and 144705. These data can be obtained free of charge via www.ccdc.cam.ac.uk/data_request/cif (or from the Cambridge Crystallographic Data Centre, 12 Union Road, Cambridge CB2 1EZ, U.K.)

X-ray oscillation photographs. Typical X-ray oscillation photographs of **1** were collected using a Bruker SMART APEX II CCD detector with graphite-monochromated Mo Kα radiation (λ = 0.71073 Å) with ω scan mode (0.3° steps). X-ray oscillation photographs of **1** at 50 K were collected using a large imaging plate area detector with a cold He gas flow installed in the BL02B1 beamline of SPring-8 (λ = 0.6874 Å). The measurements were performed using the ω scan mode (5° steps). Because of heavy sample degradation by radiation damage, full structural analyses of **1** using synchrotron radiation were not successful.

Differential scanning calorimetry measurements. Differential scanning calorimetry (DSC) measurements of single crystals of **1** (~10 mg; ground in a mortar) were carried out using a DSC3500 Sirius (NETZSCH) in a constant flow of N_2. The sample was kept in the incubator (Espec Corp. SH-221) at 25 ˚C, 97% RH for 1 day before measurements.

Impedance measurements. Impedance measurements were carried out with a Solartron SI 1260 Impedance/Gain-Phase Analyser and 1296 Dielectric Interface, in the frequency range of $1-1\times10^6$ Hz. The relative humidity and temperature were controlled by an Espec Corp. SH-221 incubator. The measurements in the low-temperature region (140−300 K) were performed using Oxford OptistatDN2 and Lakeshore 340 temperature controllers.

Solid-state ^{1}H NMR measurements. Solid-state ^{1}H nuclear magnetic resonance (NMR) measurements of **1** were performed on an AVANCE II$^+$ 400 NMR spectrometer (Bruker Biospin K. K.) with a 9.4 T UltraShield™ 400 WB superconducting magnet. ^{1}H magic angle spinning (MAS) spectra and ^{13}C cross-polarization (CP) MAS spectra were measured with a rotor of 4 mm diameter. Pulse field gradient (PFG)-NMR measurements were performed with a Diff 50 diffusion probe (Bruker Biospin K. K.).

Quantum mechanical molecular dynamics (QM-MD) simulations. The structural and dynamical properties of confined water in the nanochannels have been theoretically evaluated using divide-and-conquer density-functional tight-binding molecular dynamics simulation technique[5]. In the DC calculations, the whole system was automatically divided into several cubic grid boxes with box length of 5.0 Å and the buffer radius was set to 8.0 Å to keep accuracy and to enhance computational efficiency. The equation of motion was first integrated using the time step of 1.0 fs under canonical (*NVT*) ensemble where the temperature was maintained at 300 K using Nosé-Hoover chain thermostat[6]. After the *NVT* simulation achieving the target

temperature within 750 fs and remaining stable over the rest of equilibration up to 2.2 ps, production run was carried out for 10 ps with the reduced time step of 0.5 fs under microcanonical (*NVE*) ensemble. We have used the recent extension of DFTB method dubbed as DFTB3/3ob[7–10] augmented with Grimme's DFT-D3 (Becke-Johnson damping) dispersion correction (shortly D3(BJ))[11,12]. For platinum, for which the 3ob parameter to describe electronic and repulsive contributions of DFTB energy is not available, the parameterization of Pt-N and Pt-Br has been carried out using the DFTB+ code[13] and the automatized DFTB parameterization toolkit (ADPT)[14]. Details of parameter development and benchmark are discussed in Section 2-1. To obtain reference data for optimizing Pt-N and Pt-Br parameters, density functional theory (DFT) calculations were performed. The electronic band structures and lattice constants for bulk platinum metal were calculated using the PBEsol functional[15] and the projector augmented wave (PAW) method[16,17] with a plain-wave energy cutoff of 450 eV and a *k*-point sampling of $31 \times 31 \times 31$ as implemented in the Vienna ab-initio simulation package (VASP)[18–21]. The geometry of molecular platinum complexes was optimized using the ORCA program system[22] at the B3LYP[23,24]-D3(BJ) level of theory. The chosen basis sets for Pt and other elements were the Stuttgart effective core potential (ECP)[25] and def2-TZVPP[26], respectively.

2. Supplementary Discussions

2-1 Syntheses

Synthesis of [Pt(dach)(bpy)]$_4$(NO$_3$)$_8$ (square: □) (dach = (1R, 2R)-(–)-1,2-Diaminocyclohexane; bpy = 1,4-bipyridine)

The self-assembly reactions of cis-protected square-planar Pt^{II} complexes and linear ligands has been thoroughly studied since the first report of a molecular square complex[27–29]. These combinations yield an equilibrium of kinetically stable molecular triangles (△) and thermodynamically-stable molecule squares (□) (Supplementary Scheme 1). The starting material, Pt(dach)(NO$_3$)$_2$ was synthesized according to a published procedure[30]. Pt(dach)(NO$_3$)$_2$ (650 mg, 1.50 mmol) and bpy·2HNO$_3$ (423 mg, 1.50 mmol) were added to 70 mL of water and refluxed for 4 weeks. Here, bpy·2HNO$_3$ was employed instead of bpy to obtain a maximum yield of the square complex[29]. After filtering, the filtrate was evaporated to dryness. The obtained yellow powder was thoroughly washed with ethanol and ether to remove the HNO$_3$. The ratio of

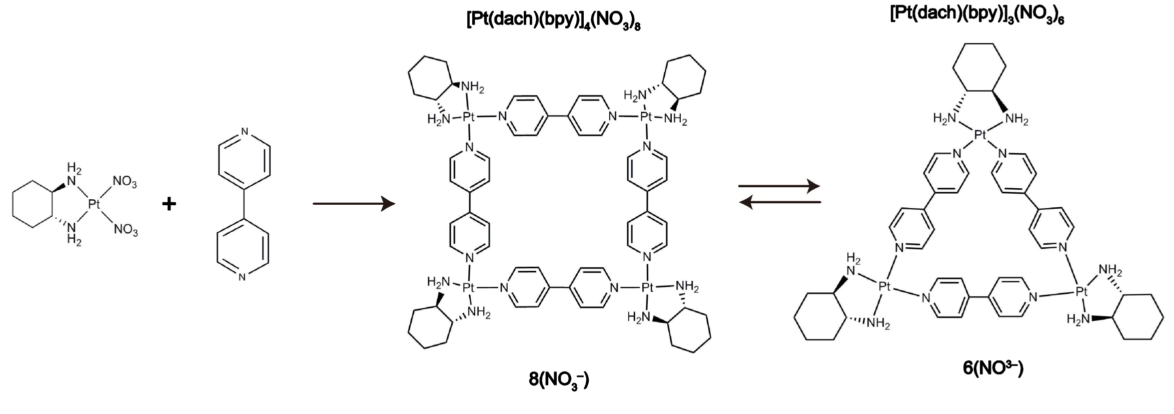

Supplementary Scheme 1 | Reaction equilibrium between square (□) and triangle (△) complexes.

square(□)/triangle(△) was estimated to be 5/1 from the ^{1}H NMR spectra (Supplementary Fig. 1).

Isolation of the square complex is possible by the following anion exchange process. Three mL of concentrated (60%) sulfuric acid was added to a concentrated aqueous solution of the mixture powder (880 mg in 25 mL H_2O) that was stirred at rt. After an excess amount of ethanol was added to the solution, the precipitate was collected by centrifugation and thoroughly washed with ethanol and ether until the wash liquid became non-acidic. The pale yellow powder (740 mg) was dried in air. The ^{1}H NMR spectra suggest that the square complex (**SO_4^{2-} salt**) was successfully isolated (Supplementary Fig. 2). The square-shaped framework was also confirmed by the single-crystal X-ray diffraction (SCXRD) of the benzenesulfonate salt (**BS^- salt**; Supplementary Fig. 6), which was obtained by the slow mixing of benzenesulfonic acid and the mixture powder in water. **BS^- salt** was not used for the synthesis of **1** because of its poor solubility. **SO_4^{2-} salt:** ^{1}H NMR (600 MHz, DMSO-d6): δ 1.14 (brs, 2H, dach-C), 1.36 (brs, 2H, dach-C), 1.57 (brs, 2H, dach-C), 1.97 (brd, 2H, dach-C), 2.48 (brs, overlapped with solvent peak, dach-C), 5.94 (brs, 2H, dach-N), 6.52 (brs, 2H, dach-N), 8.14 (d, J = 6.6, 4H, bpy), 9.04 (d, J = 6.6, 4H, bpy); Elemental analysis (%) calcd for $C_{24}H_{88}N_{16}Pt_4(HSO_4)_{6.2}(SO_4)_{0.9}{\cdot}14H_2O$: C 27.43, H 4.40, N 8.00, S 8.12; found: C 27.53, H 4.30, N 8.07, S 8.15.

Synthesis of $[Pt(dach)(bpy)Br]_4(SO_4)_4{\cdot}32H_2O$ (1)

The synthesis of metal-organic nanotube **1** was performed as shown in Supplementary Scheme 2. A methanol solution (50 mL) of the powder obtained above (**SO_4^{2-} salt**, 370 mg) was treated with an excess amount of Br_2 and stirred at rt for 10 min. The solution was evaporated to dryness and washed with a small amount of ethanol, acetone and ether to remove excess Br_2. Bromination was confirmed by ^{1}H NMR measurement (Supplementary Fig. 3). Single crystals of

the brominated square complex were obtained by the slow bromination reaction of an aqueous solution of the starting material, but these single crystals were not used for the synthesis because of their poor solubility. However, the brominated structure of the square complex could be confirmed by SCXRD (Supplementary Fig. 7). The brominated sample and non-brominated sample (370 mg) were dissolved in 40 mL of water, and 15 mL of tetrabutylannmonium sulfate solution (50wt.% in H_2O: TBA-SO_4) was added. The precipitated red-purple powder was collected and washed with ethanol. The obtained red-purple powder was dissolved in 30 mL of water again and precipitated with the addition of 15 mL of a TBA-SO_4 solution to completely remove contaminant hydrogen sulfate ions and bromide ions. The purple powder was air-dried (680 mg; yield: 60% from the starting material, Pt(dach)$(NO_3)_2$. Single crystals were obtained by slow THF diffusion to the aqueous solution of the powder sample. (over 70% yield from powder sample). Elemental analysis (%) calcd for $C_{64}H_{88}N_{16}O_{16}S_4Br_4Pt_4{\cdot}28H_2O$: C 25.04, H 4.73, N 7.30, S 4.18; found: C 24.73, H 4.35, N 7.26, S 4.25. IR and TGA spectra are shown in Supplementary Figures S4 and S5, respectively.

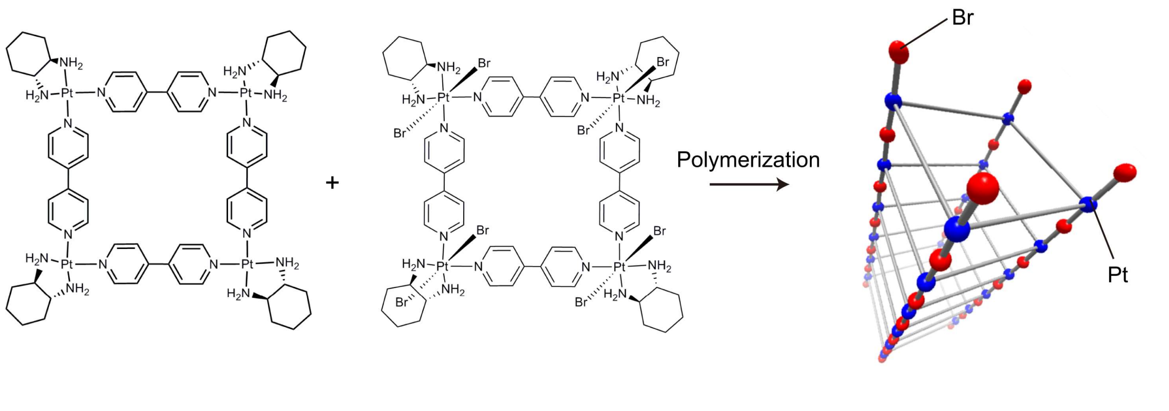

Supplementary Scheme 2 | Schematic image of constructing a tube compound.

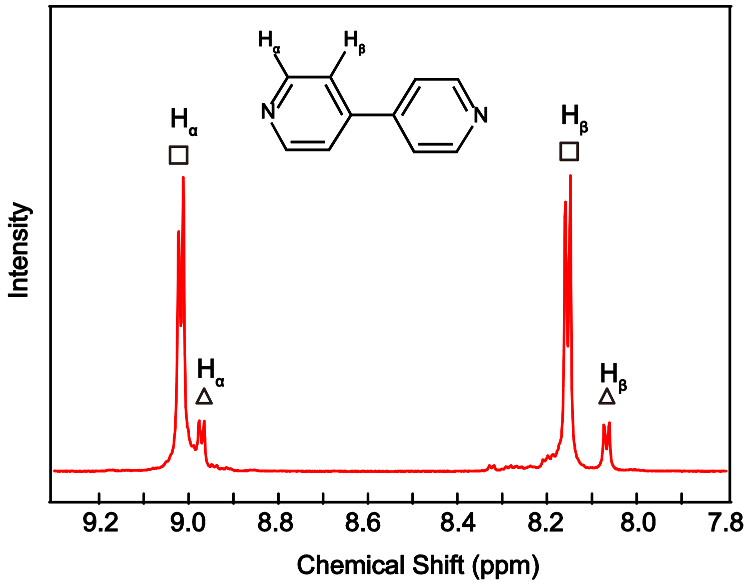

Supplementary Figure 1 | Aromatic region of the ¹H NMR spectrum (DMSO-d6 solvent) of the square (□)/triangle (△) complexes.

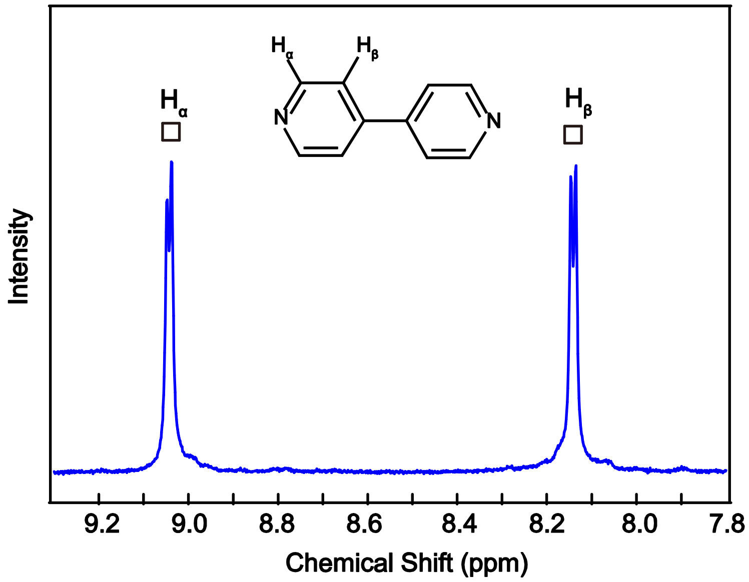

Supplementary Figure 2 | Aromatic region of the ¹H NMR spectrum (DMSO-d6 solvent) after purification by anion exchange (SO_4^{2-} salt).

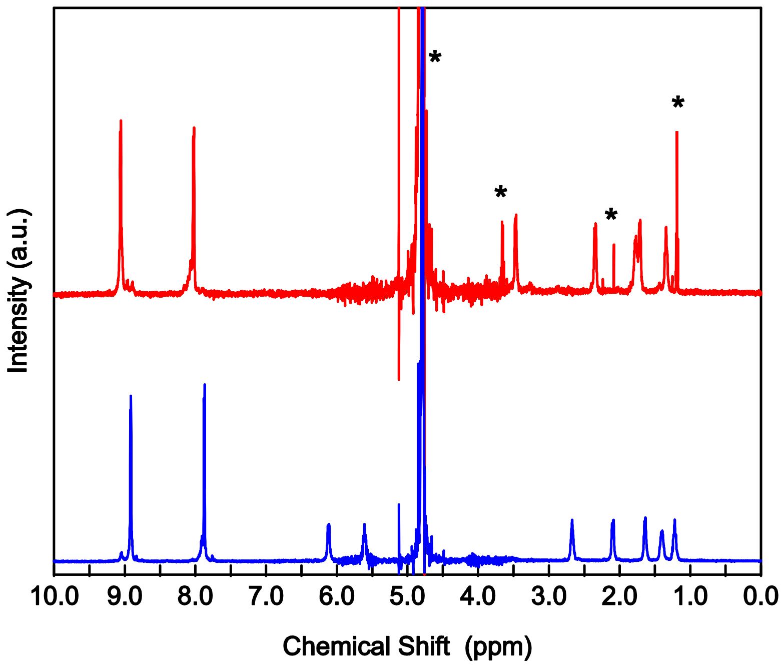

Supplementary Figure 3 | ^{1}H NMR spectra (D_2O solvent) before and after bromination. The blue line and red lines represent the spectra before and after bromination, respectively. The sharp peaks (*) at 1.17 and 3.65 ppm and 2.08 ppm come from ethanol and acetone, respectively, and the intense and broad peak (*) at 4.79 ppm is the solvent residual signal. The peaks at approximately 1.0~3.5, 5.5~6.0 and 7.5~9.0 are assigned to the C-H of dach, N-H of dach and C-H of bpy, respectively. The spectra were similar to each other, but the peaks observed for the brominated sample shifted downfield, which originates from the change of the oxidation number of the platinum. The valence state of platinum for the brominated sample is +4, and this high oxidation number would attract the electron density located at dach and bpy, resulting in a downfield shift of the ligand peaks. The peaks of dach N-H are not observed for the brominated sample, which indicates a rapid exchange of H between amines of the dach ligand and the D_2O solvent. This result is because of the high acidity of the amines of the dach ligand in the brominated square complex, which is confirmed by DFT calculations in Supplementary Fig. 29.

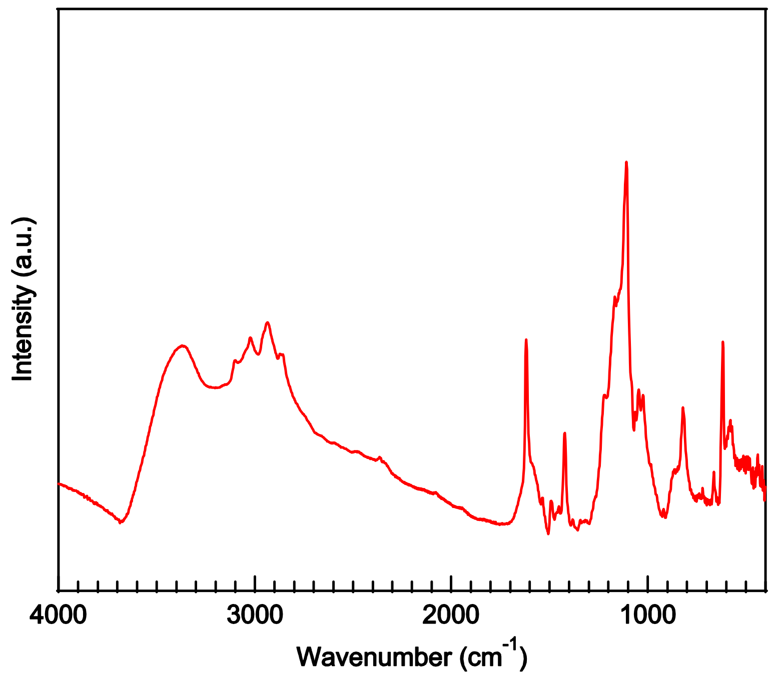

Supplementary Figure 4 | FTIR spectrum of 1. The intense peak at approximately 1100 cm^{-1} and the sharp peak at approximately 620 cm^{-1} could be assigned to the stretching mode and the bending vibration mode of SO_4^{2-}, respectively. The sharp peak at approximately 1400 and 1600 cm^{-1} could be associated to the C=C or C=N stretching modes of the bpy ring, respectively, and the peak at approximately 820 cm^{-1} is assigned as the bending vibration mode of the C-H of the bpy ring. The C-H stretching modes of the bpy and dach ligands were observed as sharp peaks at approximately 3000–3100 and 2850–2950 cm^{-1}, respectively. The very broad and intense band at approximately 3450 cm^{-1} could be assigned to the N-H stretching mode of dach and the O-H stretching mode of the crystallisation water.

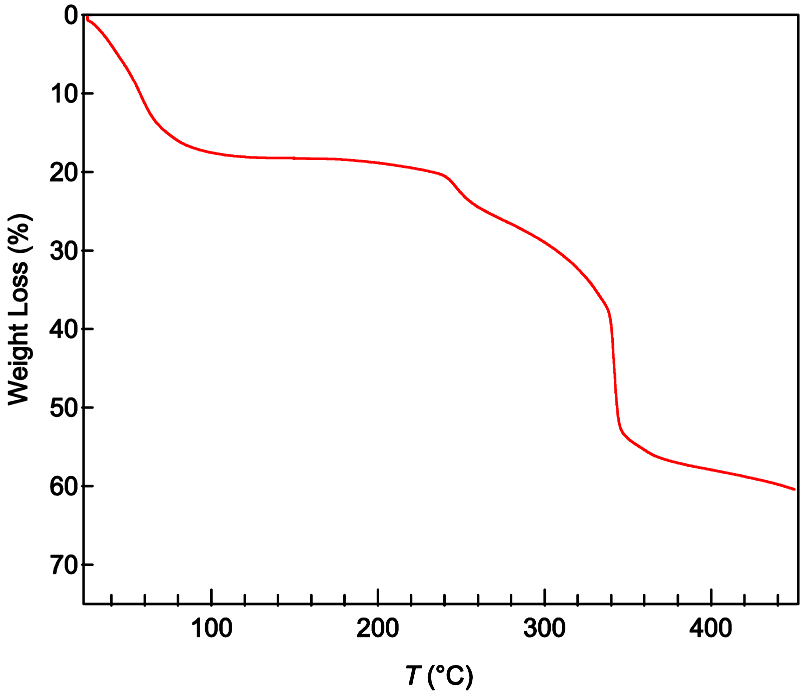

Supplementary Figure 5 | Thermogravimetric analysis of 1. The first weight loss (approximately 18%) was observed at approximately 100 ˚C due to desorption of H_2O (Calcd. 18.3% for −32H_2O). Above 230 ˚C, the decomposition of the framework was observed. The second weight loss (approximately 27%, between 230 and 270 ˚C) could be attributed to the loss of the bridging bromide (Calcd. 10.2% for −4Br), and the third (approximately 57%, between 330 and 370 ˚C) came from losses of bpy (Calcd. 19.9% for −4bpy) and dach (Calcd. 14.5% for −4dach).

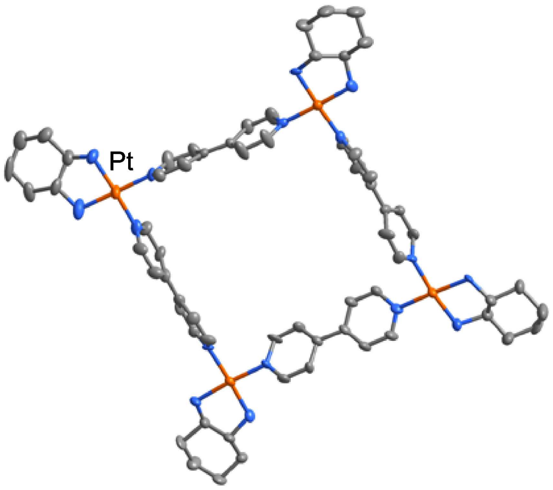

Supplementary Figure 6 | Molecular structure of divalent platinum complex [Pt(dach)(bpy)]$_4$($C_6H_5SO_3$)$_8$·8H_2O at 100 K (50% probability ellipsoids). A square-shaped structure was confirmed, where the Pt^{II}(dach) unit is coordinated by bpy in a square-planar geometry. All counter anions and crystallization water molecules are omitted for clarity. Platinum, carbon and nitrogen are shown in orange, grey and blue, respectively.

Crystallographic data: $C_{112}N_{16}O_{32}Pt_4S_8$, M_r = 3118.16, crystal dimensions 0.30 × 0.03 × 0.02 mm^3, triclinic, space group *P*1 (no.1), a = 11.1189(3) Å, b = 16.8886(5) Å, c = 18.1097(5) Å, α = 110.2680(10)°, β = 97.098(2)°, γ = 91.942(2)°, V = 3154.92(16) Å^3, Z = 1, d_{calcd} = 1.641 g cm^{-3}, T = 100(2) K, F(000) = 1480.00, μ = 4.613 mm^{-1}, 32420 unique reflections out of 79979 with $I > 2\sigma(I)$, R_{int} = 0.0302, 1075 parameters, final R factors R_1 = 0.0655 ($I > 2\sigma(I)$), wR_2 = 0.1982 (all data), goodness-of-fit (GOF) = 1.035, Flack parameter = 0.071(4), CCDC reference number: 1441697.

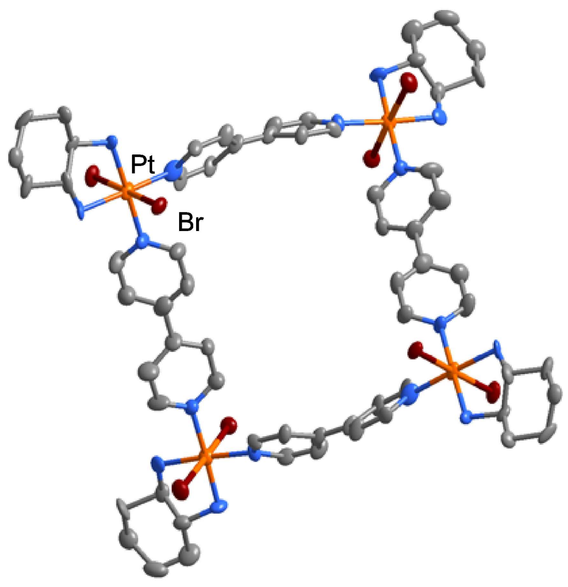

Supplementary Figure 7 | Molecular structure of tetravalent platinum complex $[Pt(dach)(bpy)Br_2]_4(Br_3)_3(NO_3)_5{\cdot}22H_2O$ at 100 K (50% probability ellipsoids). A brominated square structure was confirmed, where the $Pt^{IV}(dach)Br_2$ unit is coordinated by bpy in a square-planar geometry. The square structure was distorted, probably due to electrostatic interaction between the square framework and polybromides inside the square. All counter anions and crystallization water molecules are omitted for clarity. Platinum, bromine, carbon and nitrogen are shown in orange, red, grey and blue, respectively.

Crystallographic data: $C_{64}N_{22}O_{37}Pt_4Br_{17}$, M_r = 3807.56, crystal dimensions 0.30 × 0.06 × 0.06 mm^3, orthorhombic, space group, $P2_12_12_1$ (no.19), a = 25.9483(51) Å, b = 30.9894(61) Å, c = 7.6442(15) Å, α = 90º, β = 90º, γ = 90º, V = 6146.8(6) $Å^3$, Z = 2, d_{calcd} = 2.057 g cm^{-3}, T = 100(2) K, F(000) = 3482.00, μ = 10.136 mm^{-1}, 10850 unique reflections out of 30105 with $I > 2\sigma(I)$, R_{int} = 0.0596, 615 parameters, final R factors R_1 = 0.0809 ($I > 2\sigma(I)$), wR_2 = 0.2290 (all data), goodness-of-fit (GOF) = 1.012, Flack parameter = 0.109(8), CCDC reference number: 1441696.

2-2 Crystal structure of 1

Single crystals were prepared by the vapour diffusion method using a water solution of **1** and THF as described above. Suitable single crystals were selected in the mother liquid and quickly transferred to paraton oil to avoid undesired solvent loss. The crystallographic data are summarized in Supplementary Table 1. Supplementary Figs 8–13 show the crystal structure of **1**. CCDC reference number: 1441705. Because two sulfate anions show a highly disordered nature, some geometrical and thermal parameter restraints (DFIX, DANG, ISOR, DELU, SIMU, EADP) were used in their refinement. For the bpy ligands, a disordered structure was modelled because they have large thermal motion or no fixed orientation. Some geometrical and thermal parameter restraints (FLAT, SADI, ISOR, DELU, SIMU) were also used in their refinement. Moreover, thermal parameter restraints (ISO, DELU, SIMU) were used in the refinement on dach ligands. A thermal parameter restraint (ISO) was used in the refinement of the crystallisation water. The total accessible void volume of **1** determined by PLATON (Spek, A. L. (2009)) is 22.5% (1209 $Å^3$) per unit cell volume.

The final Flack parameter[30] (0.25(3)) indicates that the chirality is not clear, despite the presence of enantiomerically pure dach ligands in **1**. The loss of chirality during the reaction process would not occur[31]. Therefore, we suspect that the pseudo mirror plane caused this problem because the heavy Pt and Br atoms are centrosymmetrically arranged with respect to each other. These heavy atoms dominate the X-ray scattering and produce a false minimum at the inverted structure in the structure refinement[32]. The chair conformation of the dach ligands in **1** resulted in a somewhat flattened structure with vertically long thermal ellipsoids for dach carbon atoms.

Supplementary Table 1 | Crystallographic data of 1

Formula	C64 Br4 N16 O48 Pt4 S4
Formula Weight	2989.00
Temperature (K)	100(2)
Wavelength (Å)	0.71073
Crystal system	Orthorhombic
Space group	*I222* (no.23)
a (Å)	5.4559(1)
b (Å)	30.0459(8)
c (Å)	32.7895(8)
V (Å^3)	5375.1(3)
Z	2
Calcd Density (g/cm^3)	1.847
μ (mm^{-1})	6.828
F(000)	2792.00
Crystal size (mm^3)	0.30 × 0.03 × 0.03
θ_{min}, θ_{max} (°)	2.8, 28.3
Total reflections	16714
Unique reflections	5194
Parameters	400
R_{int}	0.0305
Goodness-of-fit	1.163
R_1 [I>2σ(I)]	0.0479
wR_2 (all reflection)	0.1254
max, min $\Delta\rho$ (e Å^{-3})	1.73, –1.15
Flack parameter	0.25(3)

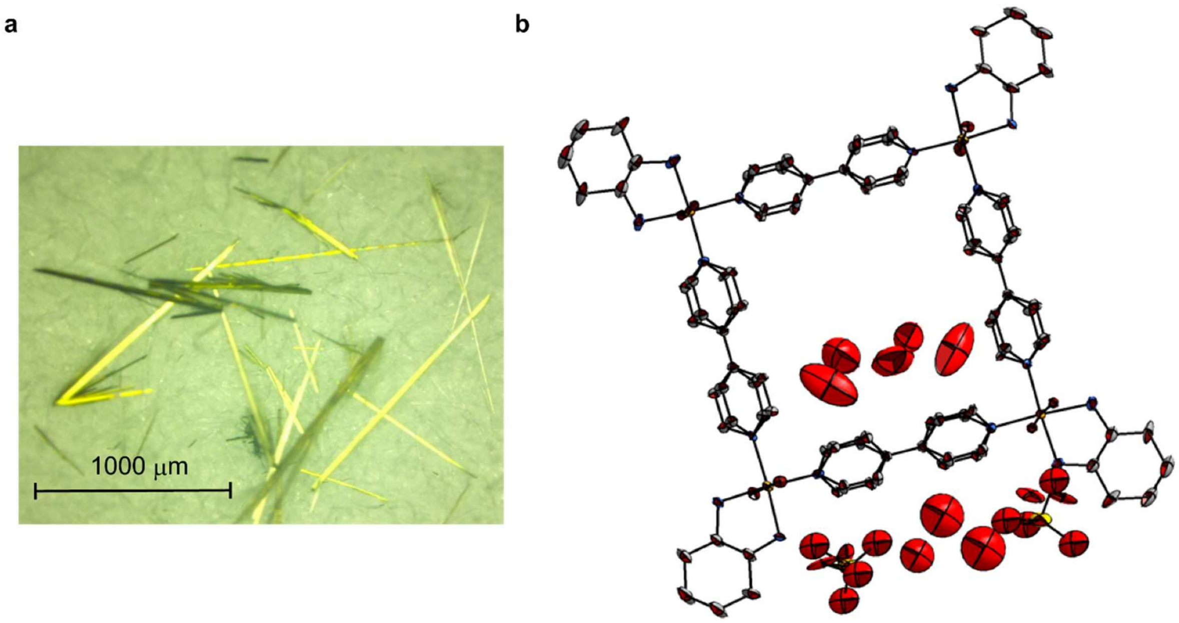

Supplementary Figure 8 | Typical single crystals and ortep-style drawing of 1. a, Needle shaped single crystals of **1** were shown. **b,** Ortep-style drawing of **1** (25% probability). There are two crystallographically independent Pt sites (Pt1 and Pt2) and Br sites (Br1 and Br2). The bpy ligands were modelled by two orientations with occupancies of 0.5. O1–O8 are oxygen atoms in sulfate anions. The occupancies of the sulfate ions were set to 0.5. O9–O13 are crystallization waters inside the tube, and O14–O20 are crystallization waters outside the tube. Because O17–O20 are crystallization waters that are located at unoccupied sulfate ion sites, their occupancies were set to 0.5. Per one tube unit, 12 crystallization waters inside the tube and 20 crystallization waters outside the tube were observed.

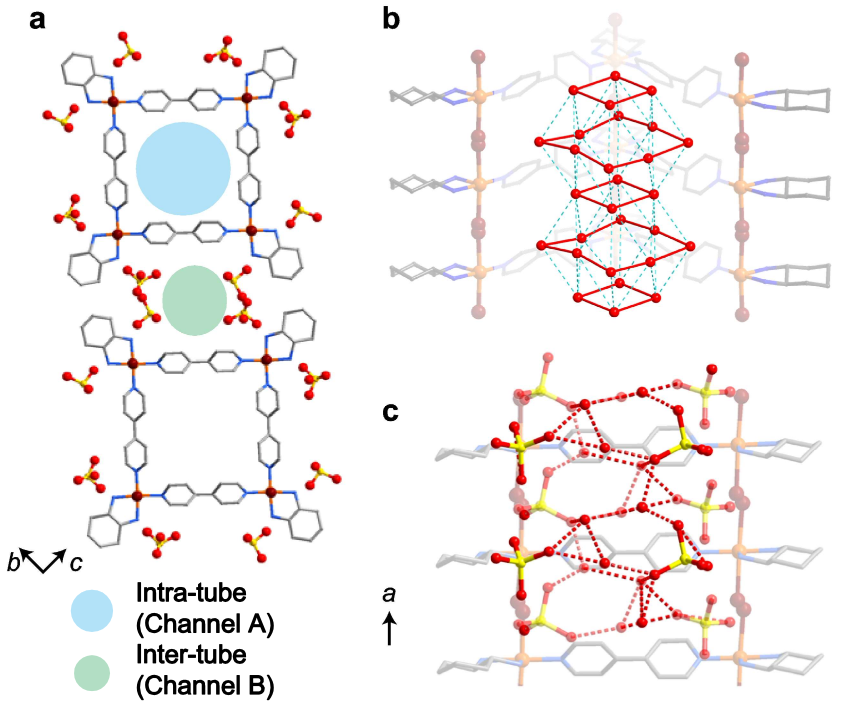

Supplementary Figure 9 | Several single-crystal structure descriptions. a, Perspective view of crystal packing along the *a* axis. Intra-tube and inter-tube nanochannels are shown by the light blue and light green colours, respectively. Both nanochannels are separated by bpy ligands, and the openings were approximately 2 Å, which are much smaller than twice the Van der Waals radius of oxygen (3 Å). **b,** Water clusters formed at the intra-tube nanochannel. Tetramer and octamer-like water clusters were observed, the former surrounded by bpy ligands, and the latter surrounded by bridging halides. These clusters would be weakly bonded by hydrogen bonds (with O–O distances of 2.9-3.4 Å: shown by light green dotted line) to form a 1D water column. **c,** Water clusters formed at inter-tube nanochannel. Crystallization waters and sulfate ions are connected by hydrogen bonds. The occupancy of the SO_4^{2-} sites is 0.5, and water molecules are expected to locate on unoccupied SO_4^{2-} sites, which would also have strong hydrogen bonds with sulfate ions or amine sites of the tube framework.

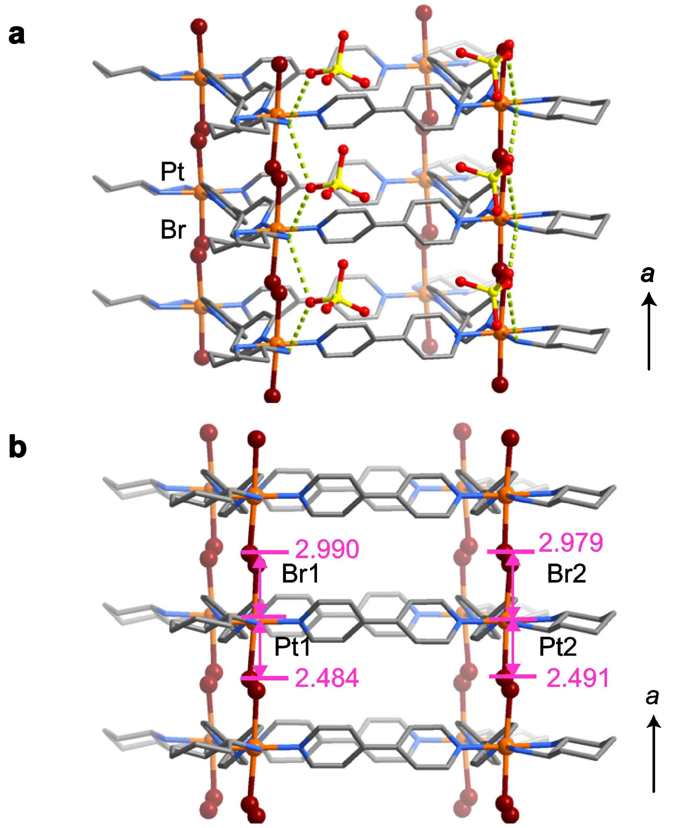

Supplementary Figure 10 | Tubular framework of 1. a, Hydrogen bond networks of SO_4^{2-} and amines of chelate ligands of **1**. Dotted lines show hydrogen bonds between SO_4^{2-} ions and amines of dach ligands. Note that the occupancy of the SO_4^{2-} sites is 0.5, and water molecules are expected to locate on unoccupied SO_4^{2-} sites. **b**, Perspective view of disordered bridging bromide ions of **1**. The bridging bromide ions are disordered with half-occupancies between two sites around the midpoints of adjacent Pt ions. The longer Pt–Br distances of Pt1–Br1 and Pt2–Br2 are 2.990 and 2.979 Å, respectively, and the shorter Pt–Br distances of Pt1–Br1 and Pt2–Br2 are 2.484 and 2.491, respectively. Disorder of bridging halide ions is often observed in the MX-chain systems because of a weak interaction between the chains[33].

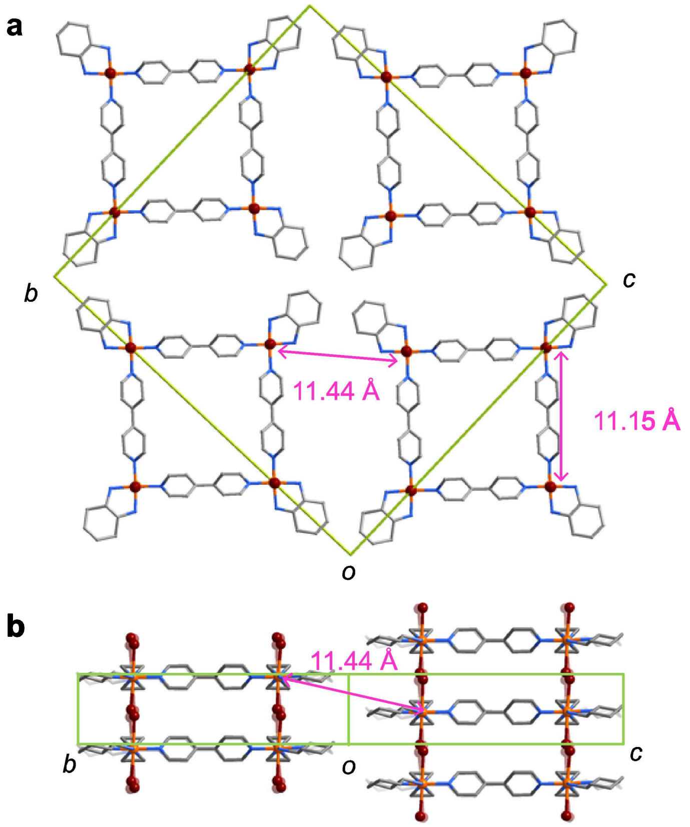

Supplementary Figure 11 | Packing structure of 1. a, Perspective view of unit cell along *a* axis. Arrows in the figure show the nearest inter- and intra-tube Pt-Pt distances of 11.44 and 11.15 Å, respectively. Compared to the inter-tube Pt–Pt distance of 8.726 Å in the previous report of the similar MX-tube compound ($[Pt(en)(bpy)I](NO_3)_8{\cdot}16H_2O$; en = ethylenediamine)[34], a longer distance was attained due to the bulky dach ligand. **b,** A perspective view of the unit cell perpendicular to the *a* axis.

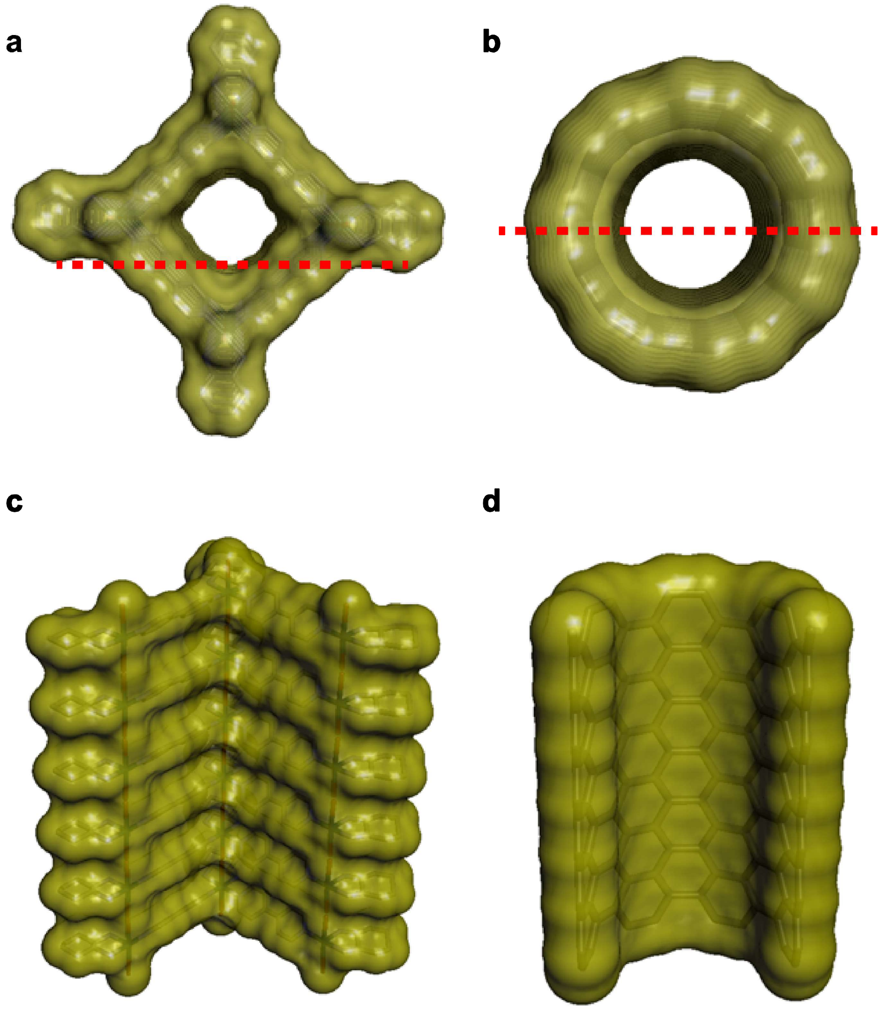

Supplementary Figure 12 | Surface shapes of hydrophobic nanochannels of 1 and the CNT. The solvent-accessible surfaces of **1** and the CNT (with a radius of *ca.* 6.0 Å) are coloured in yellow. **a,** Top view of **1**. **b,** Top view of the CNT. Red dotted lines represent the slice planes. **c,** Side view of **1** sliced on the plane shown in **a**. **d,** Side view of the CNT sliced on the plane shown in **b**. The solvent-accessible surfaces are calculated with a probe radius of 1.40 Å (a radius of O) using Discovery Studio 4.5 Visualizer[35].

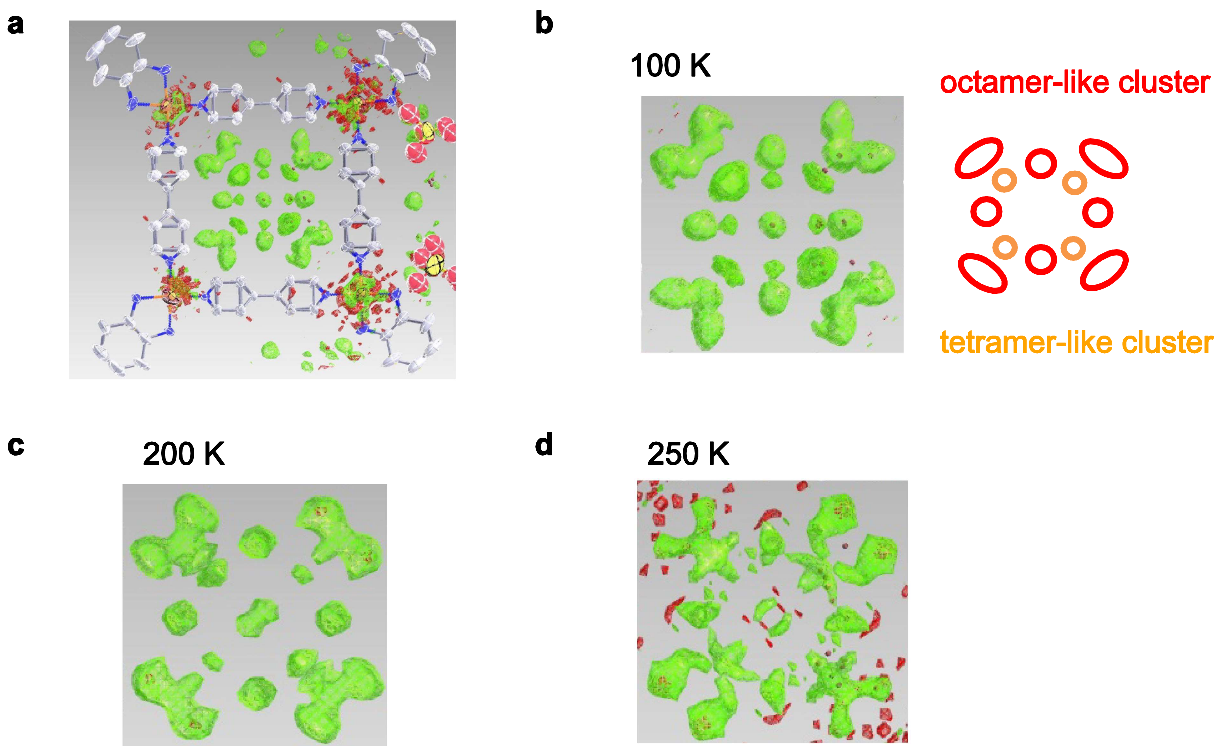

Supplementary Figure 13 | Variable temperature F_o–F_c contour maps. Counters are from –0.55 to 0.55 e A^{-3} in steps of 0.22 e A^{-3}. The counter maps were calculated from water omitted crystallographic information file using ShelXle software[36]. The electron density of water confined in channel A was visualized. **a** and **b,** counters at 100 k. **c** and **d,** counters at 200 K and 250 K, respectively. Octamer-like and tetramer-like water clusters maintained its structure at 200 K. At 250 K, the cluster structures became slightly unclear, suggesting positional disorder at 250 K. Although the low electron density was also observed at the centre of the nanochannel, we could not model water clusters.

2-3. Electronic state of 1

To investigate the electronic states of **1**, the Raman and diffuse reflectance were measured. Supplementary Fig. 15 shows the single-crystal Raman spectrum of **1** at room temperature. The intense peak of the symmetrical stretching mode ν(Pt-Br) with an overtone is clearly observed at 175 cm^{-1}. Supplementary Fig. 16 shows the diffuse reflectance spectra at room temperature. Intense and broad peaks were observed at approximately 2.2 eV, which could be assigned as the inter-valence charge transfer transition (IVCT) from Pt^{II} to adjacent Pt^{IV} sites within one MX-chain, which constitutes the four-legged tube structure. These results are strong evidence that their electronic states correspond to the charge-density-wave (CDW) state ($\cdots Pt^{II}\cdots Br–Pt^{IV}–Br\cdots$)[33], which is in good agreement with the X-ray analysis result. Diffuse scatterings with a finite line width were observed, which indicates some short-range ordered valence arrangements within the crystal (Supplementary Fig. 14). It is worth noting that in the previous report of MX-tube, $[Pt(en)(bpy)I]_4(NO_3)_8\cdot16H_2O$, a clear well-split double-peaked IVCT band was observed in the optical conductivity spectra[34]. This double-peaked band was unique among MX-type analogues and was explained by inter-tube correlations of the MX-chain because of the close inter-tube Pt-Pt distance. In the case of **1**, a single-peaked IVCT band was observed because **1** has a longer inter-tube Pt-Pt distance. In other words, by introducing a bulky dach chelate ligand, a more isolated tube system was constructed.

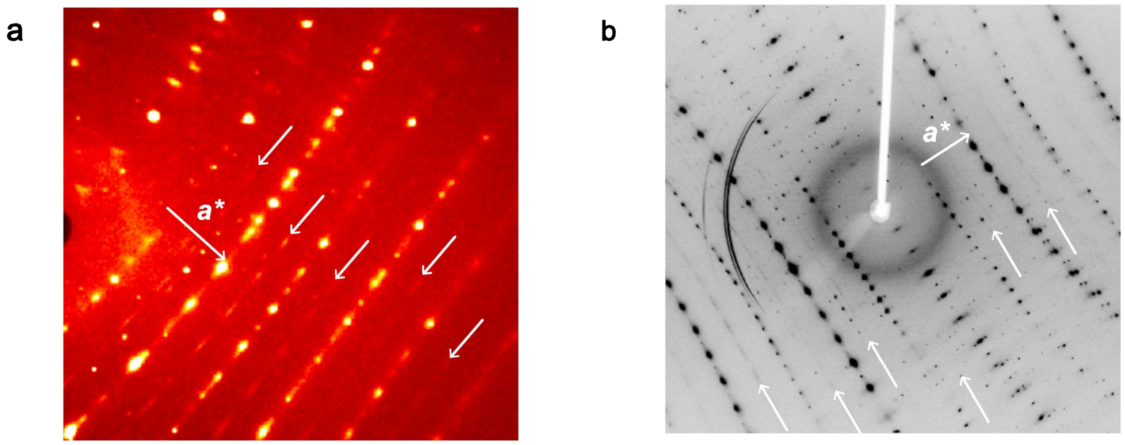

Supplementary Figure 14 | X-ray oscillation photographs of 1. a, X-ray oscillation photograph at 100 K and **b,** X-ray oscillation photograph at 50 K. The short white arrow indicates diffuse scatterings.

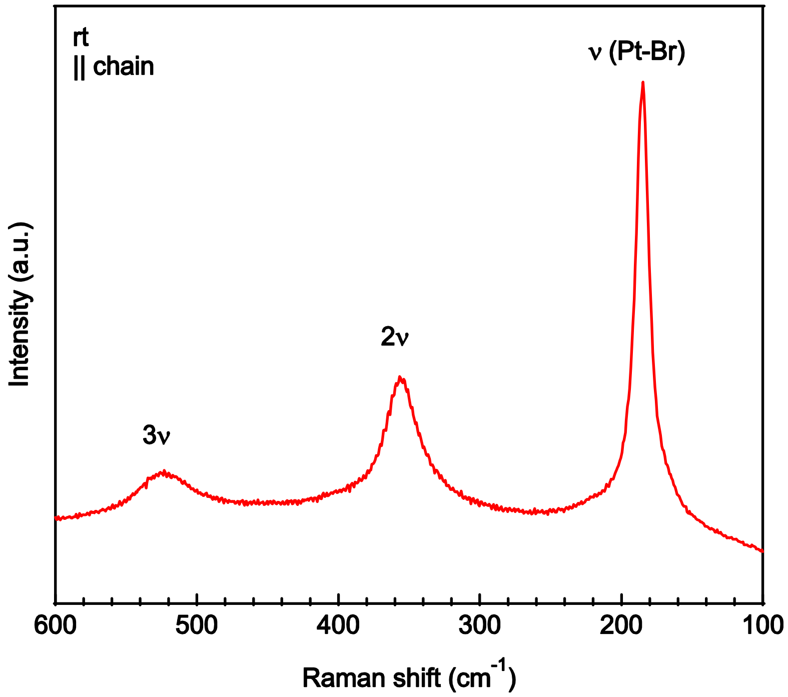

Supplementary Figure 15 | Raman spectrum of 1 at room temperature.

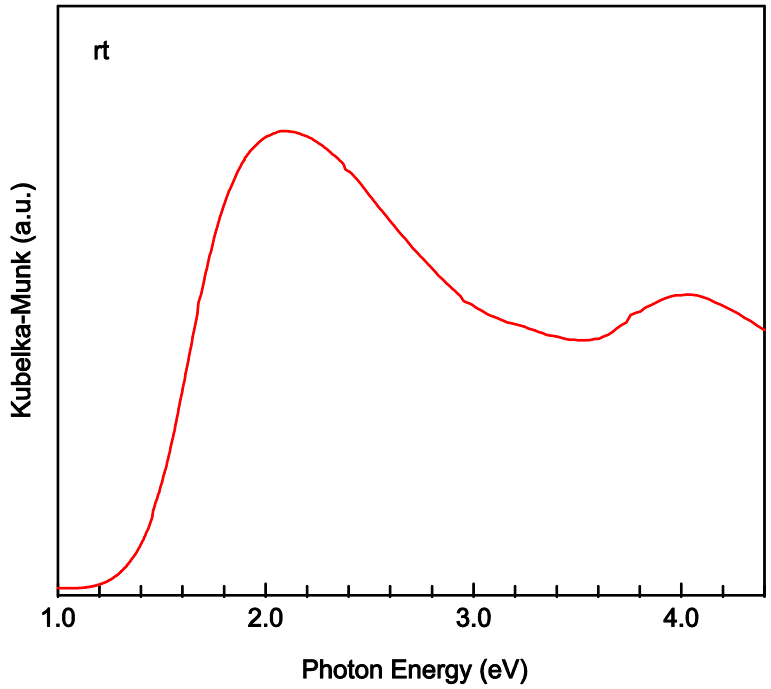

Supplementary Figure 16 | Diffuse reflectance spectrum of 1 at room temperature.

2-4. Proton conductivity measurements

Impedance measurements of a single crystal of **1** were carried out by the conventional two-probe method using gold paste and gold wires (10 μmϕ). The dimensions of the crystal were estimated under a microscope and to be 280 × 18 × 18 μm^3. The temperature dependence of the proton conductivity (95% RH) of **1** was estimated from Nyquist plots, as shown in Fig. 3 in the main text. The proton conductivity of **1** was also evaluated by using a compacted pellet sample (of 2.5 mm diameter and 0.62 mm thickness) that was prepared by pressing the powder sample (crystals ground in a mortar) at *c.a.* 400 MPa. The measurements were performed by the conventional quasi-four-probe method, using two gold wires (50 μmϕ) and gold paste. Results consistent with the single-crystal measurements were obtained (Supplementary Figs. 17−20).

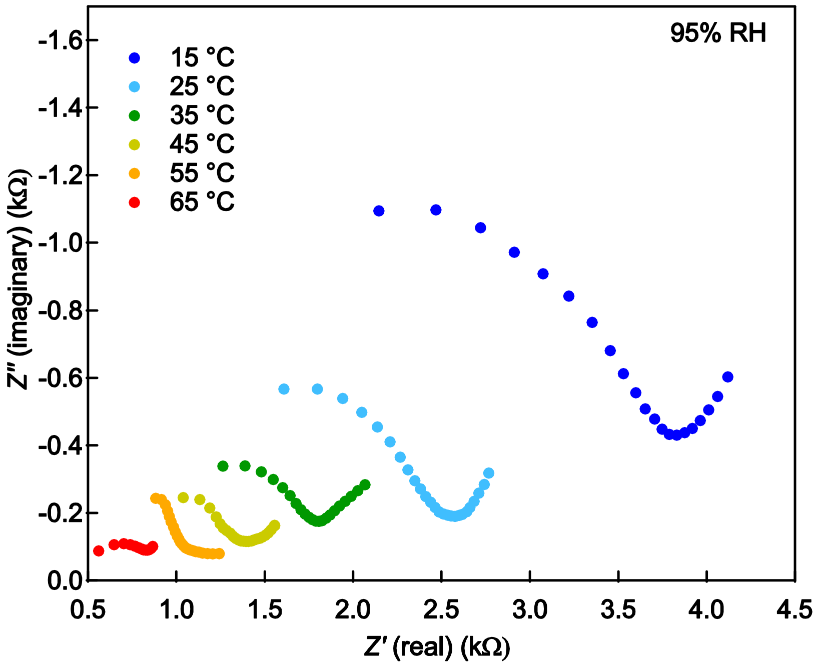

Supplementary Figure 17 | Nyquist plots of 1 (pellet) at various temperature conditions in the range of 15−65 ˚C.

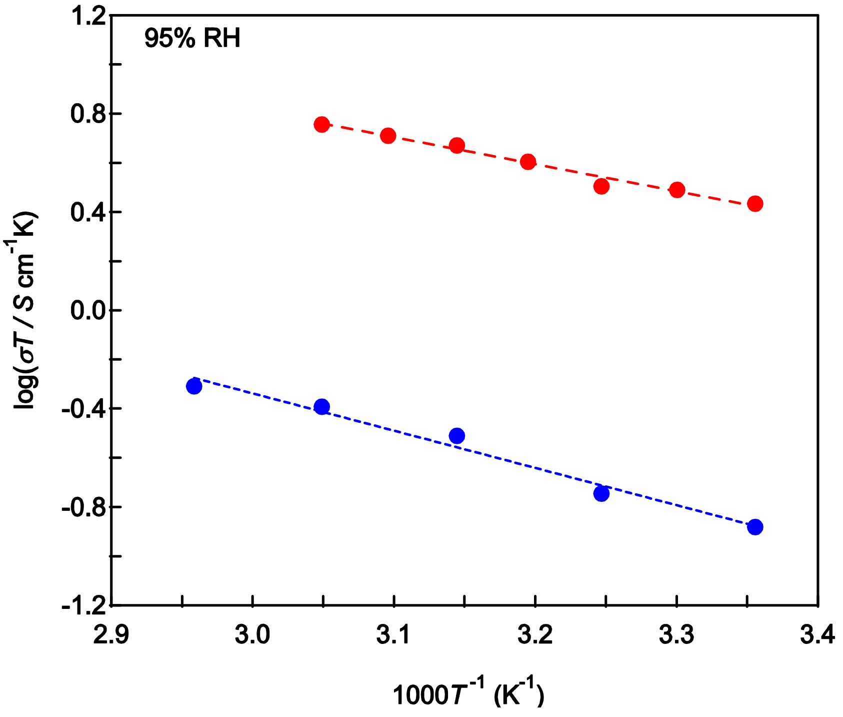

Supplementary Figure 18 | Arrhenius plots of the proton conductivity of 1 under the 95% RH condition. Red and blue circles indicate log conductivities of a single crystal sample and a pellet sample, respectively. Red and blue dotted lines indicate the least-squares fits of conductivity. The conductivity measurements were performed after equilibrating at each RH or temperature for *ca*. 4 hours. The activation energies of conductivity are estimated to be 0.22 eV for a single crystal sample and 0.29 eV for a pellet sample. The single crystal sample shows better conductivity than the pellet sample, which is consistent with previous reports[37].

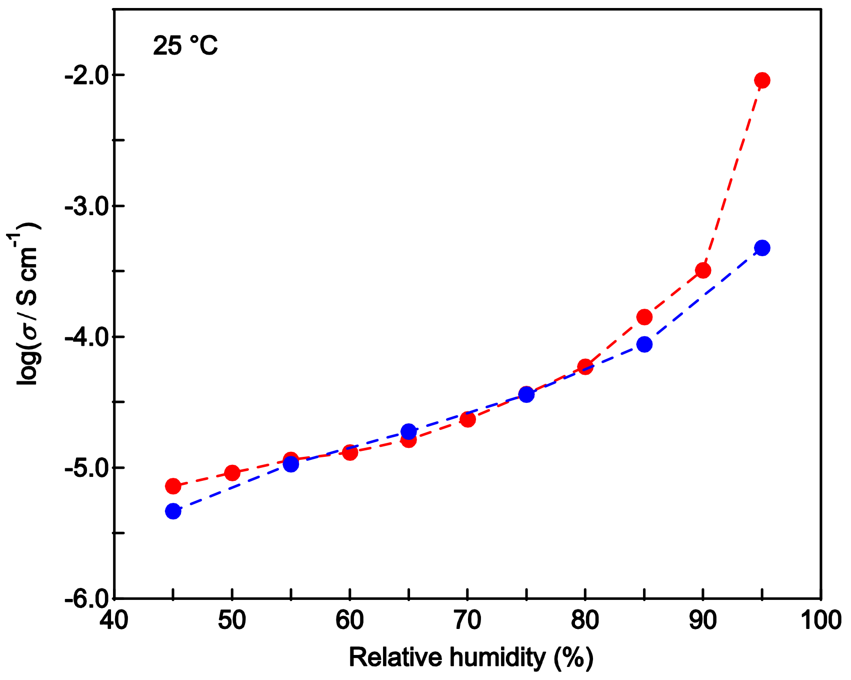

Supplementary Figure 19 | Humidity dependences of proton conductivity of 1. Red and blue circles denote log conductivities of a single crystal sample and a pellet sample, respectively. Above 85% RH, the pellet sample showed a lower conductivity than the single crystal sample. The decreased conductivity of the pellet sample under high RH was due to the anisotropy of the conductivity. However, below 85% RH, the single crystal sample showed a similar conductivity to the pellet sample. Under the low RH condition, the hydrogen bond network (proton conducting path) was fragmented, and the proton conductivity become very low, as expected from the Grotthuss mechanism, which resulted in a smaller anisotropy of conductivity.

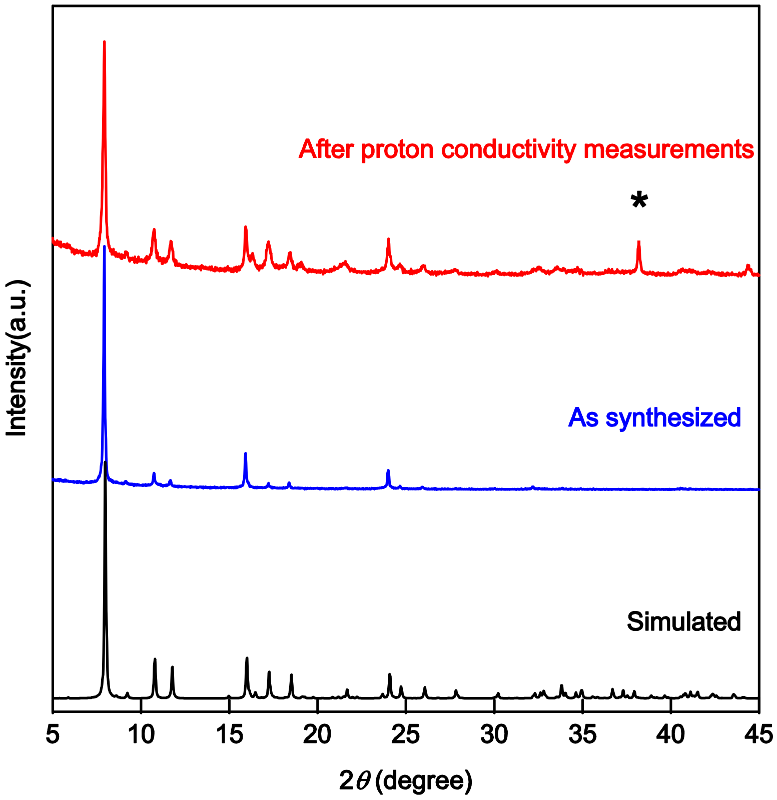

Supplementary Figure 20 | X-ray powder diffraction patterns of 1 (Cu *Kα* radiation). The black line shows the simulated pattern based on the single-crystal X-ray crystallography result (100 K). Blue and red lines show the patterns before and after impedance measurements, respectively. The sharp peak at 38 degrees in the red line (*) is assignable to Au (paste).

2-5. Solid-state ^1H NMR measurements

To check the chemical shift, ^{13}C CP MAS and ^1H MAS NMR spectra were measured. The sample was kept in an incubator at 25 ˚C, 97%RH for 1 day before measurements.

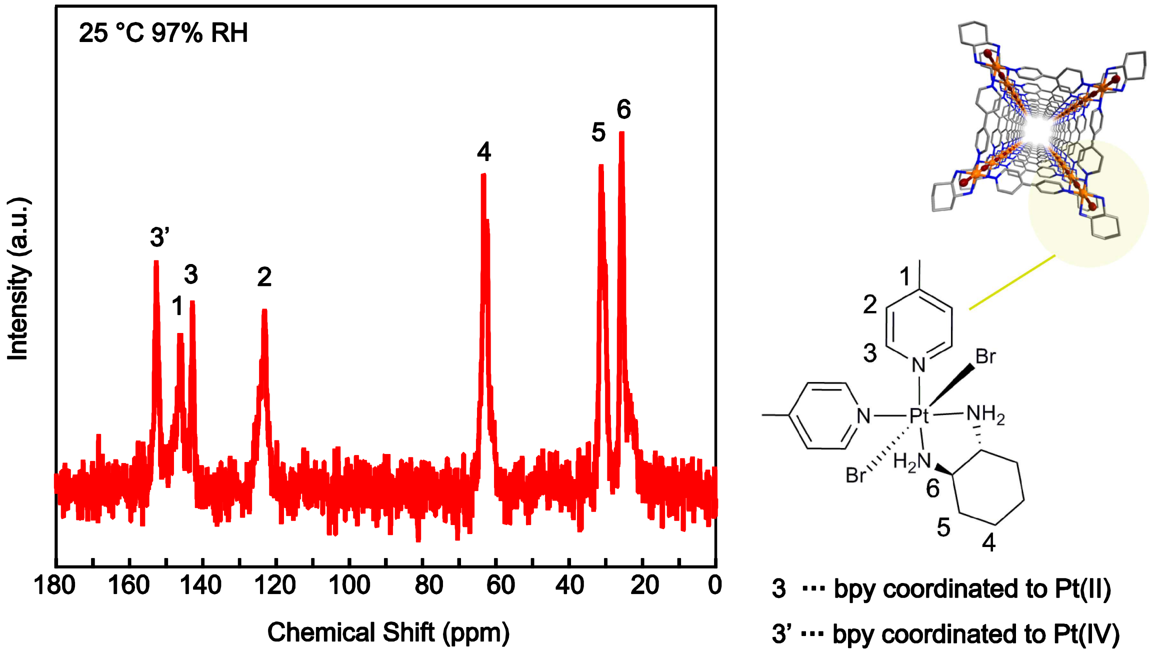

Supplementary Figure 21 | ^{13}C CP MAS spectra with a spinning rate of 10 kHz and assignment of the observed peaks. Because of the existence of Pt(II) and Pt(IV) sites, the peak of bpy (**3**) was split into **3** and **3'**.

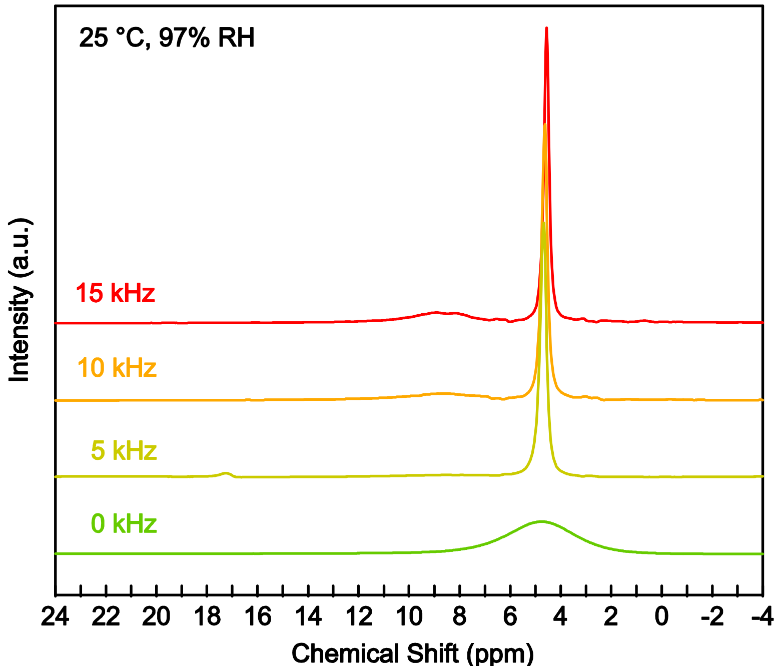

Supplementary Figure 22 | ^{1}H MAS NMR spectra with different spinning rates. The relatively sharp peak centred at 4.54 ppm and the broad peak centred at approximately 8.5 ppm can be assigned as crystallization water and the aromatic ^{1}H of bpy, respectively. Even at 15 kHz, signals from dach (at approximately 2.0 ppm for methylene ^{1}H and 6.5 ppm for amine ^{1}H) could not be observed, possibly because of the strong magnetic dipolar effect. The peak of the crystallization water of **1** (4.54 ppm) was observed slightly upfield compared to that of the bulk water (4.74 ppm). The water in an inter-tube nanochannel (channel B) is expected to shift downfield due to the acidic amine sites of **1**. On the other hand, the water in an intra-tube nanochannel (channel A) is expected to shift upfield[38]. However, the peak of the crystallization water seems to consist of a single component and is shifted upfield, indicating the exchange of a proton or water between channel A and B. The peaks were calibrated relative to an external reference of adamantane (δ = 1.91 ppm)[39].

2-6. PFG NMR measurements

To probe the molecular mobility of ^{1}H in the crystallization water of **1**, the pulse field gradient (PFG) method was performed using the stimulated spin echo sequence (Supplementary Fig. 23). Single crystals of **1** (~140 mg) were ground in a mortar and loaded into an NMR tube. After the sample was equilibrated at 25 ˚C, 95% RH in an incubator for 3 days, the tube was quickly capped. The gradient pulse strength (g) was varied in 32 steps from 0 to 25.0 T m^{-1} with a pulse width (δ) of 1 ms. The experimental diffusion time (Δ) was set to 20 ms. Figure 5a in the main page shows that the peak intensity decays with increasing g. This result demonstrates the diffusion of H_2O or its protons. The self-diffusion coefficient of ^{1}H can be calculated from the attenuation of the echo intensity, which follows different equations according to the isotropic, anisotropic, or unidirectional diffusion path. The echo decay due to the isotropic diffusion can be evaluated using Stejskal's equation[40]

$$E/E_0 = \exp(-kD) \qquad (1)$$

where $k = \gamma^2\delta^2 g^2(\Delta - \delta/3)$.

On the other hand, the decay due to anisotropic diffusion can be evaluated using the following equation[41,42]

$$E/E_0 = \exp(-kD_{\perp}) \int_0^1 \exp(-k\,[D_{\parallel} - D_{\perp}]\,x^2)dx \qquad (2)$$

where $D_{\parallel}$ and $D_{\perp}$ represent diffusion along and perpendicular to the channel alignment direction, respectively.

Unidirectional diffusion affords[43,44]

$$E/E_0 = \int_0^1 \exp(-kD_{\parallel}x^2)dx \quad (3)$$

Among the self-diffusion data so far reported for proton conductors, isotropic diffusion has been the most frequent. However, the anisotropic and unidirectional diffusion models have also been applied for materials having 1D nanochannels[42] or biological tissue[43,44].

Figure 5 in the main text shows the best fits to the experiment using eqs. 1–3. The result demonstrates that model (2) (anisotropic diffusion) is most likely. The fitting result gave self-diffusion coefficients of $D_{\parallel} = 2.9\times10^{-11}$ m^2s^{-1} and $D_{\perp} = 1.6\times10^{-12}$ m^2s^{-1}. Supplementary Figures 24 and 25 show Δ dependence of D and mean-square displacement (MSD) of ^1H, respectively. In Supplementary Figure 24, The decrease of D with increasing Δ was observed, indicating the existence of transport resistances on the diffusion path[41]. In addition, MSDs of the parallel and perpendicular component of D did not increase linearly with Δ and reached saturation, indicating the restricted nature of diffusion. This behaviour can be expected for restricted self-diffusion inside a cylinder. The saturation value can be calculated as $(1/6)l^2$ for the parallel component and $(1/8)d^2$ for the perpendicular component diffusion, where l and d are the length and diameter of the cylinder[41,42]. From the experimental saturation values, l and d were estimated to be *ca.*3 μm and 0.5 μm, respectively. The proton exchange rate between channels A and B is relevant to $D_{\perp}$ and seems to be not very fast. That may be the reason for the very broad signal of water (Fig. 5 in the main text).

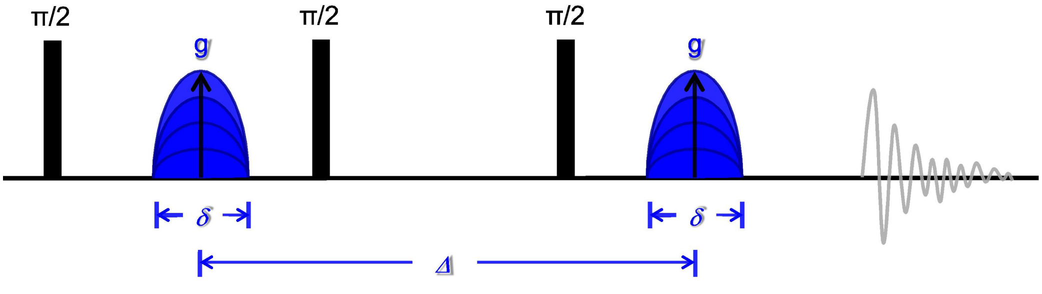

Supplementary Figure 23 | Pulsed gradient stimulated echo sequence used for the PFG NMR measurements. Here, g is the gradient field strength, δ is the pulse width and Δ is the diffusion time.

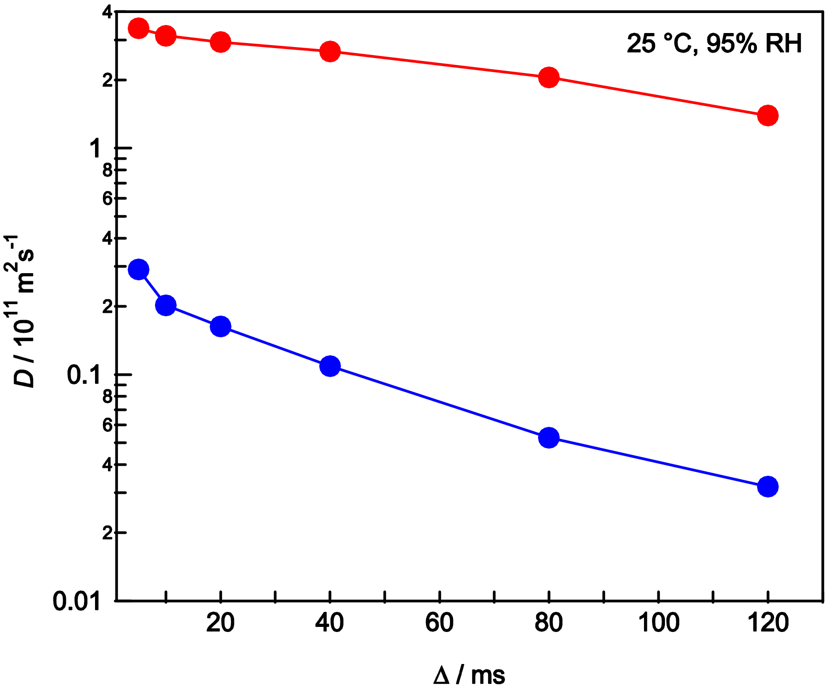

Supplementary Figure 24 | Δ dependence of *D* under 25 ˚C and 95% RH. Red and Blue lines represent $D_{\parallel}$ and $D_{\perp}$, respectively.

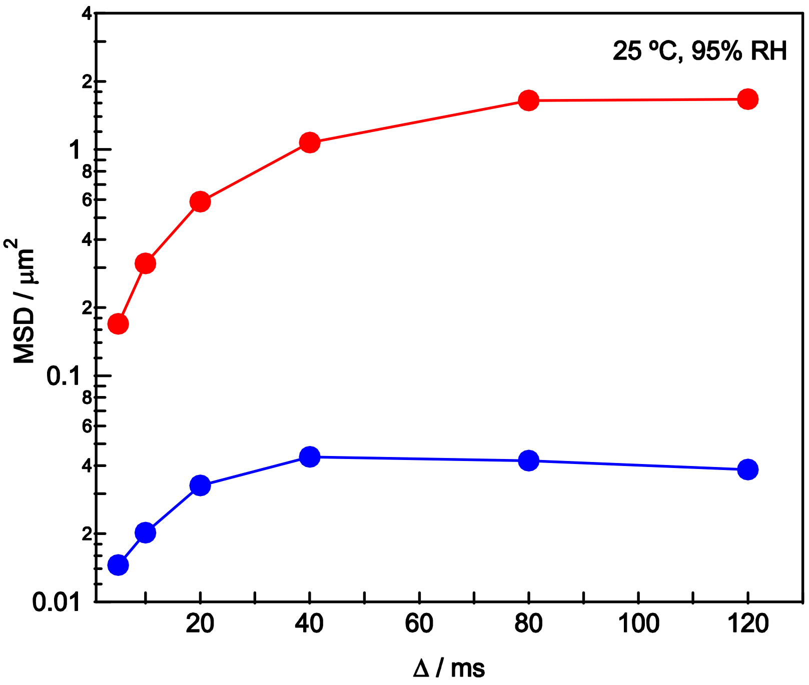

Supplementary Figure 25 | Δ dependence of mean-square displacement (MSD) of ^{1}H under 25 ˚C and 95% RH. Red and Blue lines represent MSD of $D_{\parallel}$ and $D_{\perp}$, respectively. MSD are calculated from $D \times \Delta$

Supplementary Table 2 | Δ dependence of *D* and MSD.

$\Delta/10^{-3}$s	$D_{\parallel}$ / m^2s^{-1}	$D_{\perp}$ / m^2s^{-1}	MSD ($D_{\parallel}$) / μm^2	MSD ($D_{\perp}$) / μm^2
5	3.4×10^{-11}	2.9×10^{-12}	0.17	0.015
10	3.1×10^{-11}	2.0×10^{-12}	0.31	0.020
20	2.9×10^{-11}	1.6×10^{-12}	0.59	0.033
40	2.7×10^{-11}	1.1×10^{-12}	1.1	0.043
80	2.1×10^{-11}	5.3×10^{-12}	1.6	0.042
120	1.4×10^{-11}	3.2×10^{-12}	1.7	0.038

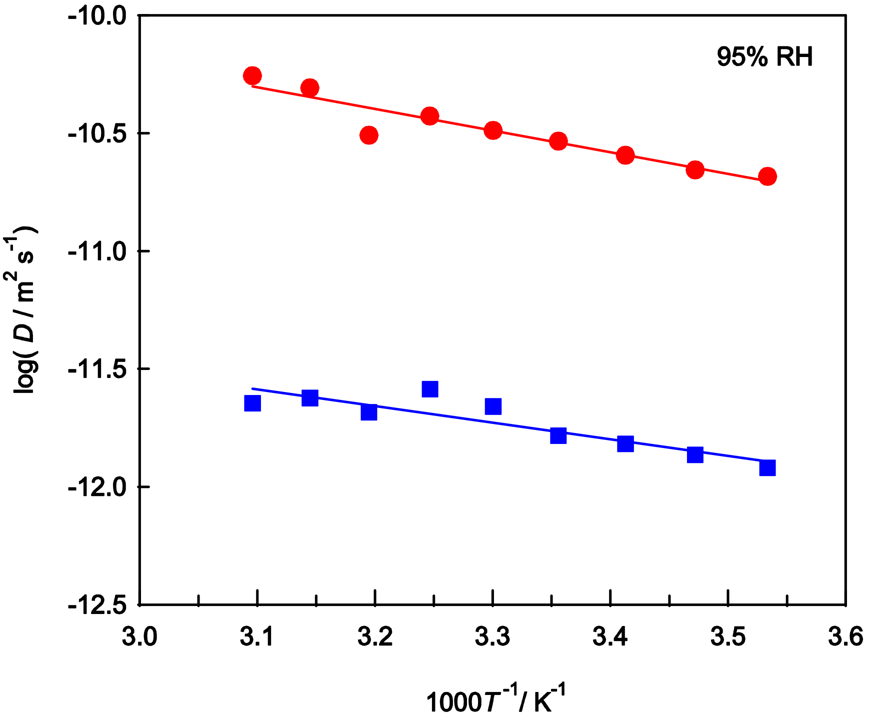

Supplementary Figure 26 | Temperature dependence of *D*. Red circles and blue squares represent log of $D_{\parallel}$ and $D_{\perp}$, respectively. Red and blue lines represent the least-squares fits of *D*. The conductivity measurements were performed after equilibrating at each temperature for *ca.* 12 hours. The activation energies are estimated to be 0.18 (±0.02) eV for $D_{\parallel}$ and 0.14 (±0.03) eV for $D_{\perp}$.

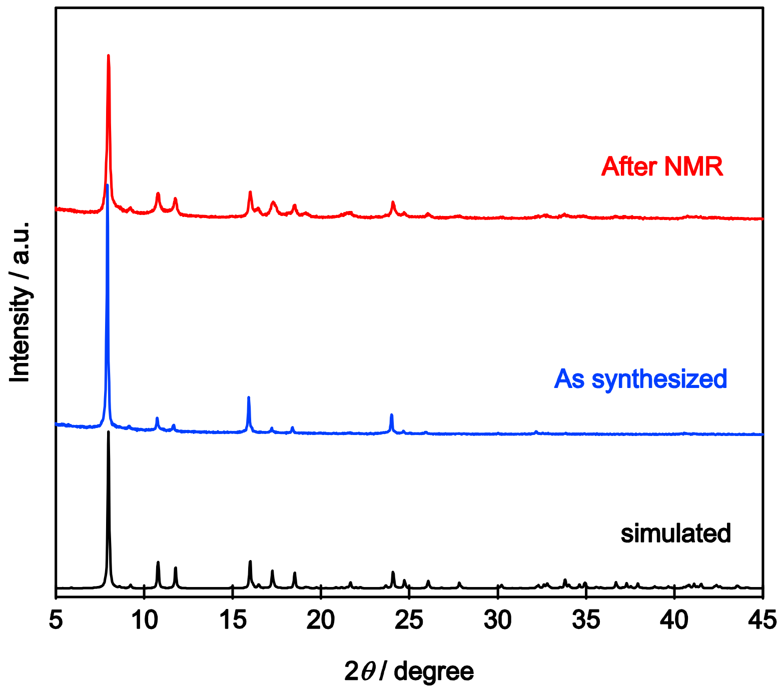

Supplementary Figure 27 | X-ray powder diffraction patterns of 1 (Cu *Kα* radiation). The black line shows the simulated pattern based on the single-crystal X-ray crystallography result (100 K). Blue and red lines show the patterns before and after solid-state ^{1}H NMR measurements, respectively. The slight loss of crystallinity is partially due to the repeated humidification in the incubator and drying in air.

2-7. H^+ dissociation energy

The acidity was evaluated as the energy required for the proton transfer from the model compound HA to an H_2O molecule, as shown below.

$$HA(aq) + H_2O(aq) + \Delta E = A^-(aq) + H_3O^+(aq). \quad (4)$$

The model structure was cut out from the crystal structure. From the orientationally disordered biphenyl moiety, the set with the least steric hindrance was chosen. The disordered dach ligand was assumed to have a chair conformation. All the hydrogen atoms were added to the crystallographically determined structure utilizing Chemcraft ver. 1.7[45]. The model structures were optimized with the coordination angle and the distance around the Pt atom frozen.

The models for the Pt^{II} and Pt^{IV} moieties are shown in Supplementary Fig. 28. The equatorial- and axial hydrogens are denoted by H1 and H2, respectively.

For the geometric optimization and single-point energy calculation for the solvated model, we employed density functional theory (B3PW91 hybrid functional), implemented in Firefly QC package[46], which is partially based on the GAMESS (US)[47] source code. For Pt and Br, the Stuttgart RSC 1997 ECP and Stuttgart RLC ECP basis sets, respectively, were applied, while for the other elements, the 6-31+G(d) basis set was used. The solvation energy was calculated with the polarizable continuum model.

Calculation of Acidity: Results

The calculated ΔE's, along with those of some acids, are listed in Supplementary Table 3. The ΔE's are in a roughly linear relationship with the experimental pK_a in water[48], as shown in

Supplementary Fig. 29. Note that all the Δ*E*'s are positive due to the products' charging energy, which is not completely compensated in the current calculation.

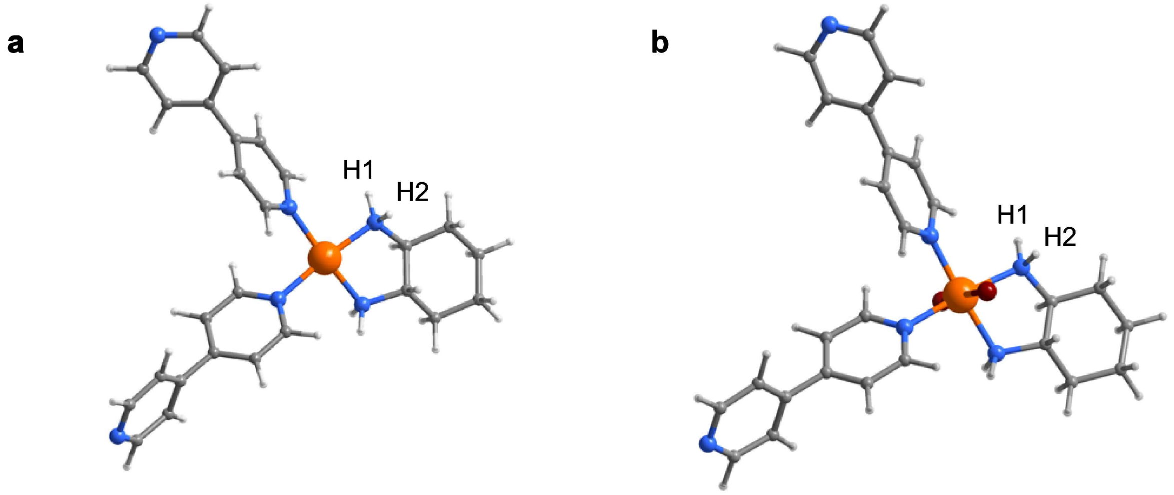

Supplementary Figure 28 | Optimized structure of the calculation model. a, Optimized structure of $Pt^{II}(dach)(bpy)_2$. **b**, Optimized structure of $Pt^{IV}(dach)(bpy)_2Br_2$.

Supplementary Table 3 | Proton-transfer energies (Δ*E*) calculated for the model structure and acids.

Molecule	Δ*E* (kJ / mol)	Molecule	Δ*E* (kJ / mol)
HCl	70.7	$Pt^{IV}(dach)(bpy)_2Br_2$	H1: 185.4
CF_3SO_3H	107.5	$Pt^{IV}(dach)(bpy)_2Br_2$	H2: 197.4
H_2SO_4	130.6	CH_3COOH	207.9
HNO_3	139.0	C_6H_5OH	236.3
$C_6H_5SO_3H$	150.2	Imidazole	253.0
CH_3SO_3H	151	$Pt^{II}(dach)(bpy)_2$	H2: 258.8
H_2CO_3	178.3	$Pt^{II}(dach)(bpy)_2$	H1: 266.3

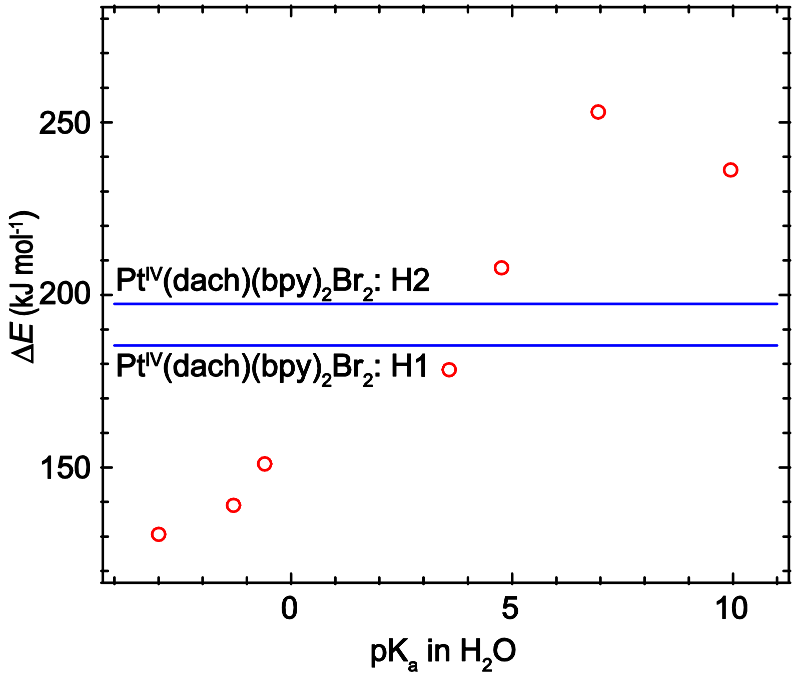

Supplementary Figure 29 | Calculated proton-transfer energy (Δ*E*) plotted against pK_a in water. The horizontal lines indicate the Δ*E*'s for the Pt^{IV} model.

2-8. DFTB parameterisation of Pt–N and Pt–Br for the nanochannel simulation

We extended the 3ob parameter set[13–15] to platinum while keeping compatibility with already existing elements. The DFTB method[12,49], which is an approximate form of DFT, requires one-centre and two-centre quantities to describe electronic structure of the system. The one-centre quantities include free atom orbital energies ε_i (i = s, p, or d orbital), Hubbard parameter (or alternatively atomic chemical hardness) U, and the first derivative of the Hubbard parameter U'. The two-centre quantities are the precomputed Hamiltonian and overlap integrals determined for each distinct atom pair. The quality of the integrals depends on the choice of pseudo atomic orbitals and reference density that can be obtained by solving atomic Kohn-Sham equation including additional confining potentials with Slater-type basis functions. The electronic parameters of Pt with valence shell occupation number n_i are given in Supplementary Table 4. Most of them have been numerically computed using the PBE functional[50] with the zeroth-order regular approximation (ZORA) for relativistic correction[51]. Note that ε_p, orbital confinement, and density confinement have been optimized to improve the overall performance using the ADPT toolkit[14].

The distance-dependent repulsive potentials are another necessary parameter for completing the DFTB total energy evaluation. The repulsive potential in general covers the first neighbour interaction and such a short-range character allows us to restrict development targets to only Pt-N and Pt-Br pairs for the nanochannel model system. The potential is represented using spline functions whose coefficients can be determined using the erepfit code[52] integrated in the ADPT toolkit. Supplementary Table 5 shows several selected platinum complexes used for the fitting of repulsive potential. Two different oxidation states of Pt(II) and Pt(IV) which may encounter during practical simulations are considered.

The created Pt electronic parameter is verified by investigating the shape of electronic band structures of the bulk Pt metal for four different phases: face-centred cubic (FCC), body-centred cubic (BCC), hexagonal close-packed (HCP), and simple cubic. As shown in Supplementary Fig. 30, the reasonable agreement with the reference DFT results is observed. We also confirmed that the present electronic parameter can meet with the repulsive potential reproducing the lattice constants of bulk Pt metals fairly well.

The optimized Pt–N and Pt–Br repulsive potentials are presented in Supplementary Table 6. The potential consists of five segments of fourth-order polynomials and the contribution are vanished beyond 4.6 and 5.3 atomic units for Pt–N and Pt–Br pairs, respectively. The following are the mean absolute deviations of bond length compared to B3LYP-D3(BJ) optimized geometries for the training set in Supplementary Table 2: 0.035 Å for Pt–Br, 0.020 Å for Pt–N, and 0.013 Å for all other types of bonds. We have then tested the DFTB3-D3(BJ) energy minimization of the MX-tube system. The model system was constructed based on the experimental XRD structure. There are two intra-tube nanochannels A and four inter-tube nanochannels B in the unit cell of $21.824 \times 30.046 \times 32.790$ Å^3. The number of confined water molecules per nanochannel is 48 for channel A and 40 for channel B, leading to 2336 atoms in total. Supplementary Fig. 31 shows the relaxed structure where the average values of optimized bond length are 2.469 and 3.023 Å for Pt-Br and 2.083 Å for Pt-N. The results of Pt-Br bond length are consistent with experimental observations of 2.48–2.49 and 2.98–2.99 Å.

The validity of the developed Pt–N and Pt–Br parameters against the nanochannel simulations is assessed through calculating radial distribution function (RDF) of Pt–Br and Pt–N pairs. The peak positions shown in Supplementary Fig. 32 are located at almost the same positions as those of optimized bond lengths (2.48 and 3.05 Å for Pt–Br and 2.09 Å for Pt–N),

hence no significant change of nanochannel spaces is ensured. All benchmarks make us confident that the developed Pt–N and Pt–Br parameters are reliable for the current purpose.

Supplementary Table 4 | DFTB electronic parameters for Pt.*

Parameter	Value
ε_s, ε_p, ε_d	−0.20428, 0.1, −0.22840
n_s, n_p, n_d	1.0, 0.0, 9.0
U, U'	0.2463, −0.08457
Basis set coefficients	0.5, 1.767, 6.245, 22.071, 78.000
Orbital confinement	3.0
Density confinement	10.0
Confinement order	4

*All numbers are given in atomic units if not unitless.

Supplementary Table 5 | Reference molecular platinum complexes used for repulsive potential fitting.*

Pt-N	Pt-Br
$Br_2Pt(II)(dien)$	$BrPt(II)CH_3$
$Br_2Pt(IV)(dach)(py)_2]^{2+}$	$Br_2Pt(II)(dien)$
$[Br_2Pt(IV)(dach)(bpy)_2]^{2+}$	$Br_2Pt(II)$
$Br_2Pt(II)(dach)$	$[Br_2Pt(IV)(dach)(py)_2]^{2+}$
$[(NH_3)_2Pt(II)(mal)]^{2+}$	$[Br_2Pt(IV)(dach)(bpy)_2]^{2+}$
$[(NH_3)_4Pt(II)]^{2+}$	$Br_2Pt(II)(dach)$
	$[Br_4Pt(II)]^{2-}$

*dien: diethylenetriamine, py: pyridine, dach: (1R,2R)-1,2-diaminocyclohexane, bpy: 4,4'-bipyridine, mal: malonic acid.

Supplementary Table 6 | Optimized Pt–N and Pt–Br repulsive potentials in fourth-order spline representation.

Pt-N

```
Spline4
5 4.6
4.89458 15.3411 -0.095351
3.40000 3.67498 1.770627087053E-01 -1.333351169352E+00 3.263098643788E+00 -2.851691156448E+00   0.000000000000E+00
3.67498 3.98568 -2.140019891221E-03 -1.856564488670E-01 9.106042526616E-01 -2.851691156448E+00   3.353228268843E+00
3.98568 4.22092 -2.620172290678E-02 -4.337085353765E-02 1.947428034996E-01 1.315656055871E+00  -3.088386787066E+00
4.22092 4.46375 -1.795815333763E-02 1.058543665920E-01 9.779061791689E-02 -1.590414973469E+00   2.509202787512E+00
4.46375 4.60000 -5.354852602551E-04 1.572042556496E-02 -1.730662342314E-01 8.467941930363E-01  -1.553726832990E+00
```

Pt-Br

```
Spline4
5 5.3
14.4879 38.3779 -1.6286
2.80000 2.94448 -1.516484095689E+00 -1.624267440217E+00 1.176611459129E+01 -1.055081493140E+01   4.197151074237E-02
2.94448 3.30271 -1.537350878127E+00 1.115425910807E+00 7.198273182998E+00 -1.052655900586E+01   3.202658921895E+00
3.30271 3.55048 -6.451955337233E-01 2.809032433998E+00 -1.648602358765E+00 -5.937371316051E+00   8.359840652791E+00
3.55048 4.52806 -1.092183034332E-01 1.407248812583E+00 -2.982668064955E+00 2.347801854301E+00  -6.452451496389E-01
4.52806 5.30000 2.016127945594E-02 -1.044707513661E-01 2.030030741882E-01 -1.753185996653E-01    5.677859469285E-02
```

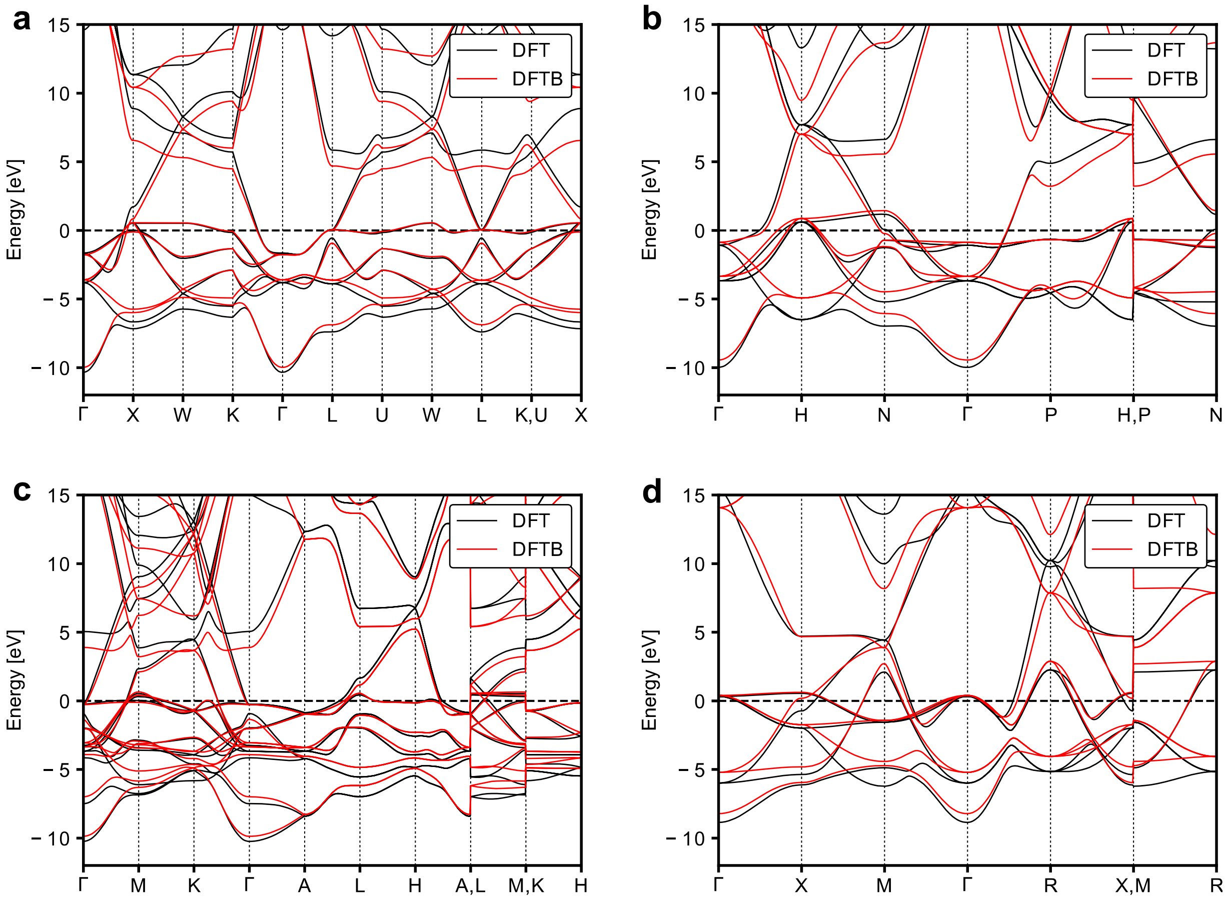

Supplementary Figure 30 | Electronic band structures of four phases of bulk Pt metals. a, FCC. **b**, BCC. **c,** HCP. **d,** Simple cubic. The red solid lines are obtained with the optimized DFTB parameter and compared with the black solid lines obtained with DFT (PBEsol functional).

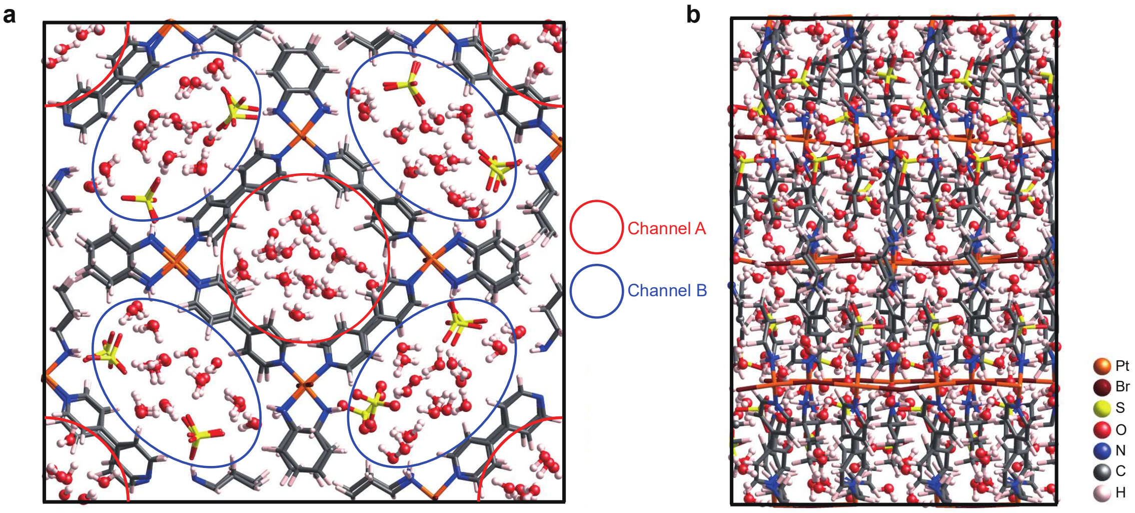

Supplementary Figure 31 | DFTB optimized structure of the MX-tube model system. a, Front view. Locations of channel A and B are indicated by black and red ellipsoids. **b,** side view. MX-tube units and SO_4^{2-} ions are drawn by stick model. Ball and stick model is used for water molecules situated in channels A and B, which are highlighted by red and blue circles, respectively (see panel **a**).

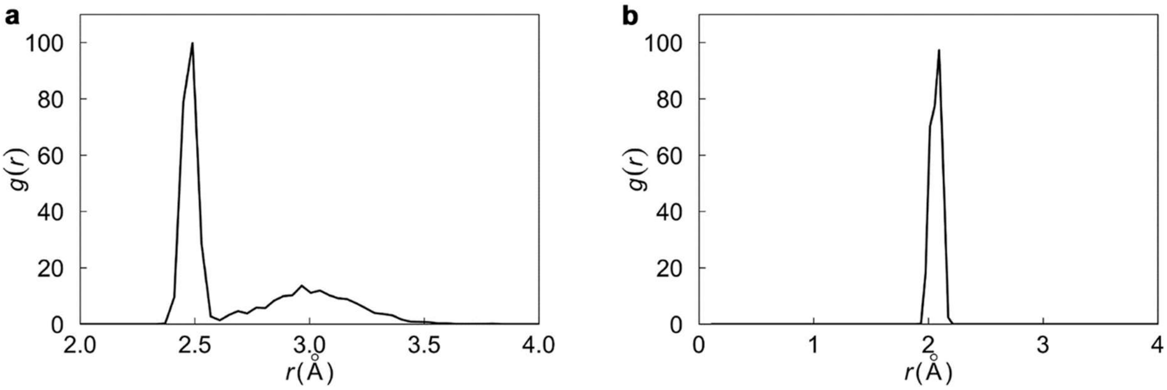

Supplementary Figure 32 | Radial distribution functions obtained from QM-MD simulation of the MX-tube system. a, Pt–Br pair. **b,** Pt–N pair.

2-9. QM-MD simulation of confined water in hydrophobic nanochannel

For proton transfer simulation using the MX-tube model system shown in Supplementary Fig. 31, one proton was randomly abstracted from amino-group and then added to oxygen in each of channel A.

Supplementary Fig. 33 shows the radial distribution function (RDF) of O–O and O–H pairs in nanochannels A and B. The corresponding results of bulk water are also plotted for comparison. The close agreement of the first peak position of O–O RDF indicates that there is no elongation or shrinkage of the first solvation shell inside the nanochannels relative to the bulk system. More structured second solvation shell seems to exist in channel A. In channel B, less pronounced second peak may attribute to the interaction with sulfate anions. Regarding O–H RDF, the first and second solvation peak positions are similar to each other, while the characteristic difference between nanochannels and bulk water is observed as the shoulder at around 5.5 Å.

The dynamical properties of proton diffusion were evaluated using the similar analyses to the previous studies for bulk water[53,54]. Specifically, vehicular diffusion coefficient (D_v) is calculated from the slope of time course changes of mean square displacement (MSD). The one-dimensional displacement along the MX-tube axis gives the parallel contribution of D_v ($D_v^{\parallel}$) and the perpendicular one ($D_v^{\perp}$) is obtained with displacement in the radial direction. The diffusion constant due to Grotthuss shuttling event (D_G) can be related with the number of protons hopping during the simulation, which is quantified as follows

$$h(t) = h(t - \Delta t) + \Delta h(t) , \qquad (6)$$

where $h(t)$ is set to zero at the beginning of the simulation and the value fluctuates at each successive time step according to $\Delta h(t)$ given by

$$\Delta h(t) = \begin{cases} 0 & \text{(if no proton jumps)} \\ -1 & \text{(if proton jumps to the last oxygen)} \\ 1 & \text{(if proton jumps to a new oxygen)} \end{cases} . \qquad (7)$$

The slope of Eq. 6 as a function of time corresponds to the proton transfer rate r_p and it is in proportion to D_G after multiplying a factor accounting for the magnitude of proton transfer length[54,55]. The overall proton diffusion coefficient (D_p) is defined as the summation of D_v and D_G.

The vehicular diffusion of confined water inside the nanochannels is first discussed. Supplementary Fig. 34 shows time-course changes of MSD for oxygen atoms in channel A, channel B, and the whole system. The corresponding results of hydrogen atoms are provided in Supplementary Fig. 35. The first 2 ps of MSD indicates the existence of transient behaviour between ballistic motion and diffusive motion in the beginning of trajectory, hence we calculated D_v from the last 8 ps of the MSD plots. As pointed out in the main manuscript, water molecules in channel A move approximately 1.5 times faster than those in channel B. The interaction with sulfate anions seems to suppress the translational motion of water molecules in channel B. The larger D_v value of hydrogen than that of oxygen in channel A indicates the existence of the Grotthuss structural diffusion. The presence of an excess proton in channel A enhances the hydrogen atom diffusivity, because of its contribution to the structural diffusion process. When the proton moves to channel B as a hydronium ion, it interacts with sulfate anion to make a "special pair dance"[56], hence the hydrogen atom moves together with the oxygen atom of water molecule.

The parallel and perpendicular components of diffusion coefficients and the ratio $D_v^{\parallel}/D_v^{\perp}$ are given in Supplementary Table 7. The diffusivity of confined water inside the nanochannels is not completely homogeneous. The calculated ratio in channel A is smaller than unity, namely the migration of proton carrier from channel A to channel B is a possible event. In channel B, water molecules tend to move parallel to the tube axis rather than the radial direction because of the presence of sulfate anions. The different favourability of diffusion direction in each channel determines the overall ratio as 1.08 and 1.17 for hydrogen and oxygen atoms, respectively.

As shown in Supplementary Table 8, the calculated D_G value is approximately 10 times larger than D_v, hence the Grotthuss shuttling has a great contribution to proton diffusion of confined water. In the trajectory, the migration of the hydronium ions initially present in each of the channel A to distinct channel B, triggered by the hydrogen bond network involving sulfate anions, have been observed at 0.6 and 4.2 ps. The hydronium ions in channel B have formed a strong interaction with sulfate anions that effectively suppresses the structural diffusivity of hydronium ion. In addition to the ordinary proton transfers between water molecules, proton transfers through a neutral [(H_2O–$HOSO_2OH$–OH_2)] complex have been also observed. The r_p and D_G values for channel A and B have been estimated from the 4.2 and 9.4 ps trajectory of hydronium ions, respectively. For more details, see also Supplementary Movies 1–3.

Considering our solid-state NMR and QM-MD simulation results, there is a discrepancy in absolute value of estimated proton diffusion coefficient between experiment and simulation. In fact, the simulated diffusion coefficients show much higher values than the experimental results. This difference would be derived from the following reasons: (1) The present simulation assumes the introduction of an excess proton in a small limited space, giving a much higher proton concentration than in normal water. This gives rise to enhance hydrogen diffusivity. (2)

The calculation of Grotthuss diffusion was performed by the accumulation of one-dimensional scalar distances (Supplementary Fig. 35), while NMR analysis gave the one-dimensional projection of the diffusion coefficient for three-dimensionally randomly jumping protons. The latter inevitably shows slower motion. (3) The calculation follows hydrogen motions up to a short time period of 10 ps. On the other hand, NMR observation is carried out in 10 to 10^2 times longer time scale, where the gradient of proton displacement giving the diffusion coefficient is expected become smaller (Supplementary Fig. 35).

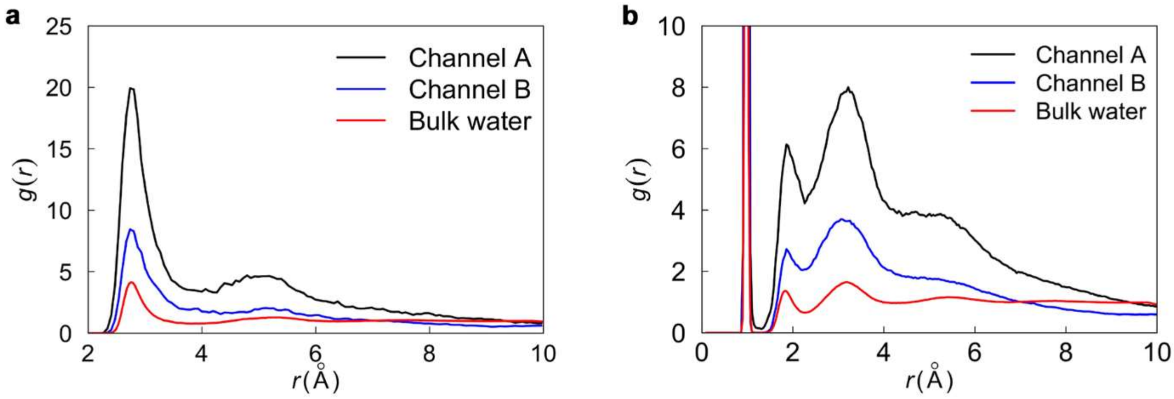

Supplementary Figure 33 | Radial distribution functions (RDF) obtained from QM-MD simulation of the MX-tube system. a, O–O pair. **b,** O–H pair.

Supplementary Table 7 | Calculated vehicular diffusion coefficients (m^2s^{-1}) and its ratio obtained from QM-MD simulation of the MX-tube model system.

	Hydrogen			Oxygen		
	Whole system	Channel A	Channel B	Whole system	Channel A	Channel B
D_v	1.3×10^{-9}	1.8×10^{-9}	1.1×10^{-9}	1.3×10^{-9}	1.6×10^{-9}	1.1×10^{-9}
$D_v^{\parallel}$	1.4×10^{-9}	1.5×10^{-9}	1.3×10^{-9}	1.4×10^{-9}	1.5×10^{-9}	1.3×10^{-9}
$D_v^{\perp}$	1.3×10^{-9}	1.9×10^{-9}	9.0×10^{-10}	1.2×10^{-9}	1.7×10^{-9}	1.0×10^{-9}
$D_v^{\parallel}/D_v^{\perp}$	1.08	0.79	1.44	1.17	0.88	1.30

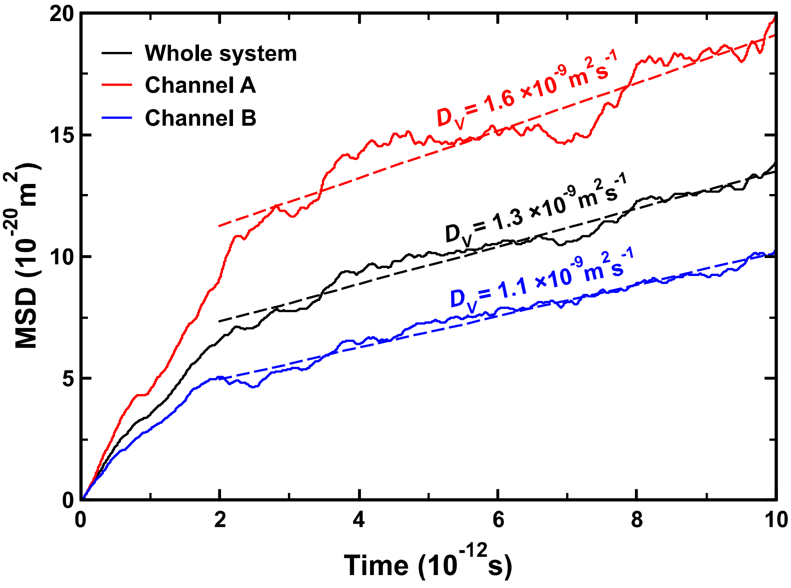

Supplementary Figure 34 | Time course changes of mean square displacement (MSD) for oxygen atoms. The dashed lines indicate the least-square fit of MSD which leads to the vehicular diffusion coefficient (D_v).

Supplementary Table 8 | Calculated and experimental diffusion coefficients (m^2s^{-1}) of water molecules in different chemical environments.

	Calculated			Experiment
	Nanochannel of **1**	Liquid (water)	Solid (ice)	Liquid (water)
D_v	1.3×10^{-9}	3.0×10^{-9}	0.0	$2.3 \times 10^{-9\dagger}$
D_G	$1.1 \times 10^{-8*}$	6.5×10^{-9}	1.9×10^{-8}	$7.0 \times 10^{-9\ddagger}$
D_p	1.3×10^{-8}	9.5×10^{-9}	1.9×10^{-8}	$9.4 \times 10^{-9\S}$

[*]Average of hydronium 1 and 2 shown in Supplementary Fig. 35b. [†]Ref. 57, [‡]Ref. 58, [§]Ref. 59.

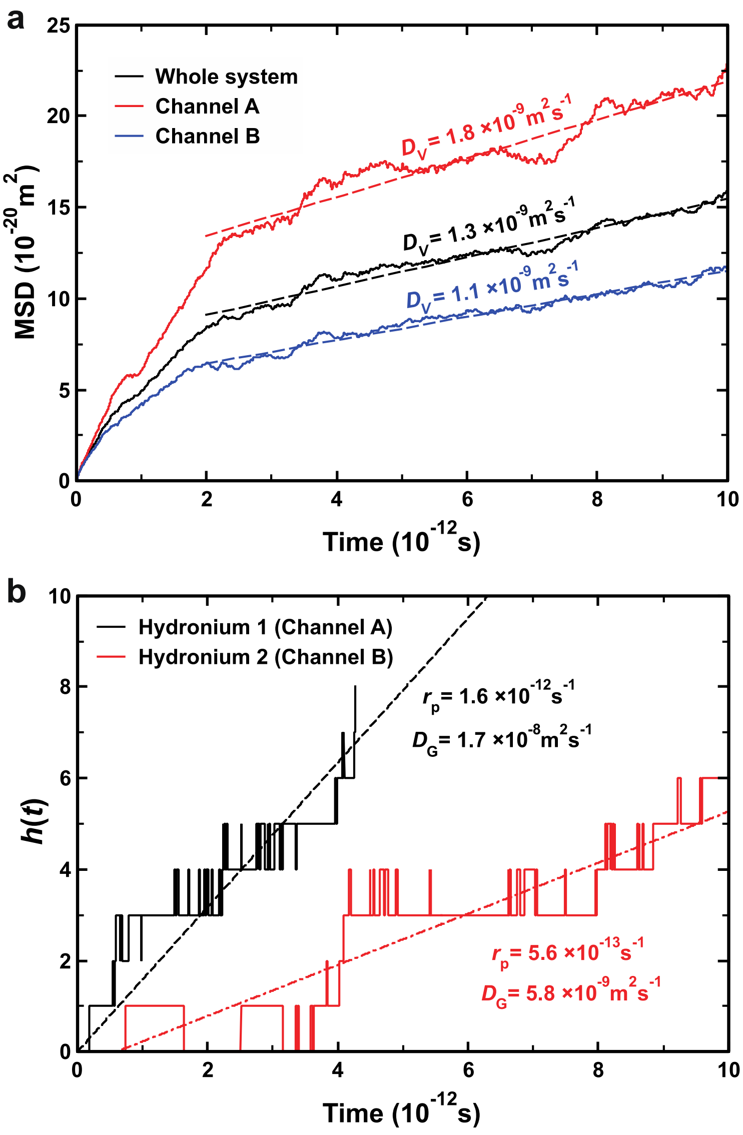

Supplementary Figure 35 | Time course changes of dynamical indices obtained from QM-MD simulations. a, Mean square displacement (MSD) of hydrogen atoms. The dashed lines indicate the least-square fit of MSD which leads to the vehicular diffusion coefficient (D_V). **b,** Proton shuttling function $h(t)$ (see Supplementary Discussion for the definition). The dashed lines indicate the least-square fit of $h(t)$ which leads to the proton transfer rate (r_p). The calculated Grotthuss diffusion coefficients (D_G) are also shown in the figure.

Details of Supplementary Movies

Models used for the movies. In supplementary movies, the MX-tube unit is represented by transparent stick model. The sulfate counter ions are represented by transparent ball and stick model. Water molecules inside the channels A and B are represented by opaque stick model. The transparent blue sphere is used for marking the most-positive-charged oxygen atom of all water molecules. All oxygen and hydrogen atoms that within 4 Å radius to the transparent blue sphere are represented by ball and stick model dynamically for clarity.

Supplementary Movie 1 | The trajectory of hydronium 1 (within channel A) from 0 to 1.5 ps in orthographic view.

Supplementary Movie 2 | The trajectory that the hydronium 1 migrates from channel A to channel B from 3.0 to 6.33 ps. The movie during the migration (around 4.2 ps) has been created using slow motion (10 times of the number of frames, or 0.1×) for clarity.

Supplementary Movie 3 | The trajectory of hydronium 2 (within channel B) from 7.5 to 9 ps in orthographic view.

2-10. Liquid-to-solid transition of confined waters

The liquid-to-solid phase transition behaviour of confined waters in **1** was investigated from DSC, solid-state ^{1}H NMR and proton conductivity measurements at low temperature (Supplementary Figs. 36–39).

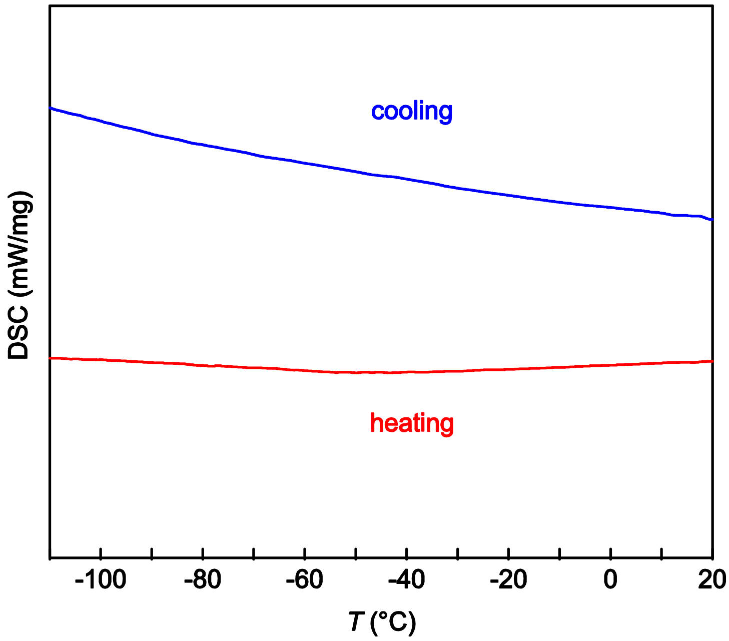

Supplementary Figure 36 | DSC curves of 1. Blue and red lines show the cooling and heating processes, respectively. The sample was kept in the incubator at 25 ˚C and 97% RH for 1 day and then hermetically sealed in an aluminium pan before the measurements. The heating (cooling) rate was set to 3 K / min (–3 K / min). The faster scan rate (10 K / min) using DSC3100SA (NETZSCH) also detected no apparent peak.

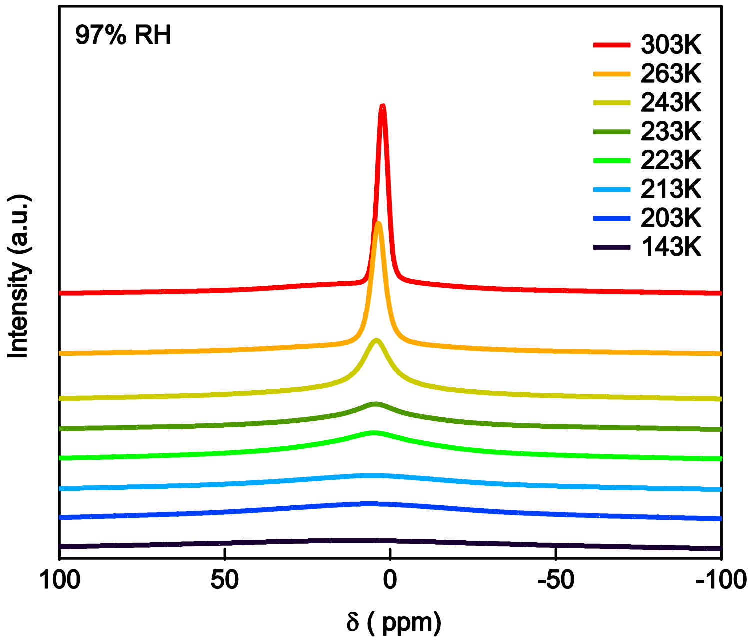

Supplementary Figure 37 | Temperature dependence of ^{1}H NMR spectra. After the sample (crystalline powder) was equilibrated at 25 ˚C, 97% RH in an incubator for 3 days, the NMR tube (ϕ = 5 mm) was quickly sealed. The measurements were performed with decreasing temperature. At each temperature, about one hour was allowed for equilibration. Measurements were conducted in 400 MHz NMR.

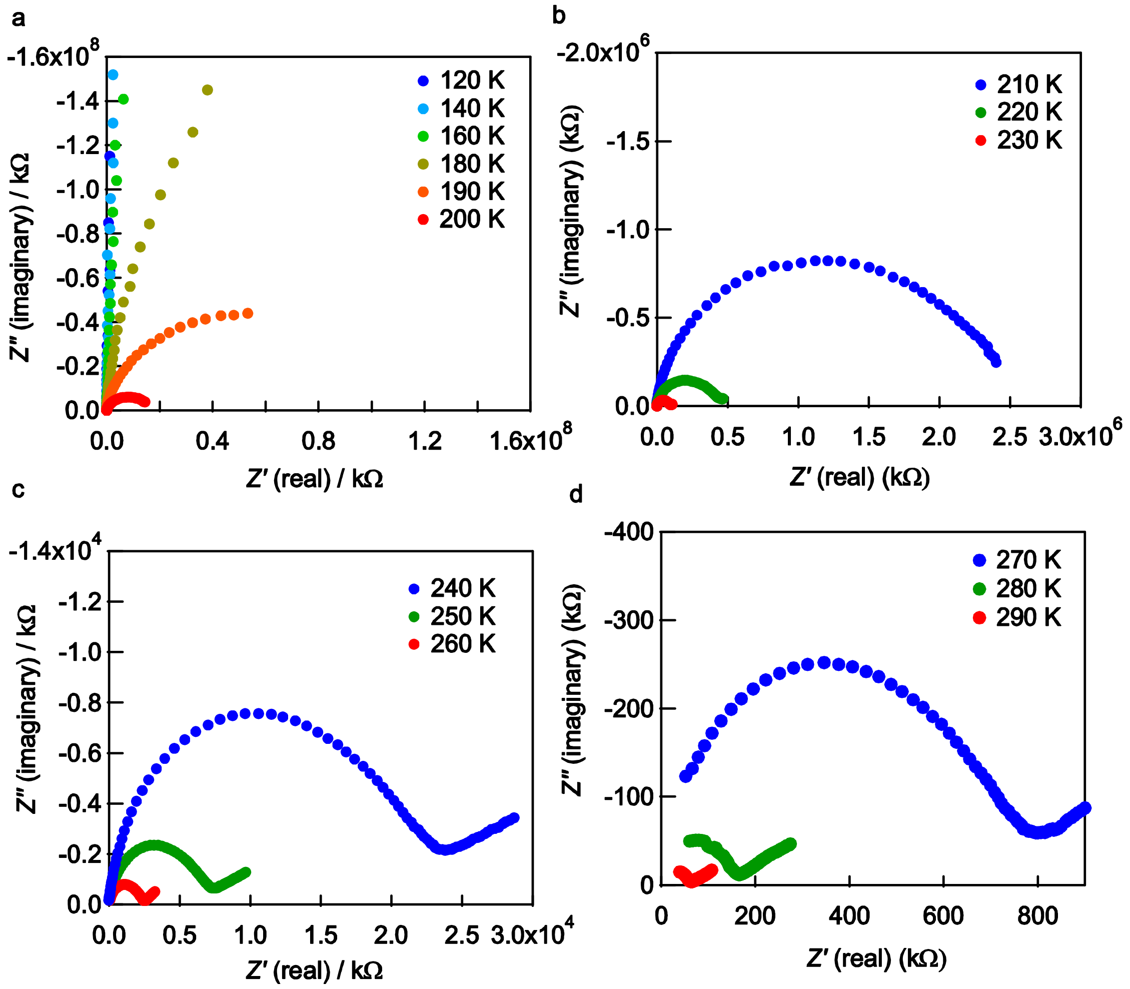

Supplementary Figure 38 | Temperature dependence of Nyquist plots in the low-temperature region. The measurements of a compacted pellet sample (with 2.5 mm in diameter, 1.38 mm thickness) were conducted by the conventional two-probe method, using two gold wires (50 μmϕ) and gold paste in a cryostat under a dry nitrogen atmosphere. To minimize sample drying, the side of the sample was covered by Apiezon grease after equilibration at 25 ˚C, 95% RH in the incubator for 1 day. At each temperature, ~1 hour was allowed for equilibration. Measurements were performed with increasing temperature. The conductivity at room temperature was smaller than that in Supplementary Fig. 19 due to the sample drying during the measurements.

For further investigation of behaviours of confined water below room temperature, variable temperature ^{1}H MAS NMR measurement was also conducted with the spin rate of 10 kHz in 400 MHz NMR (Supplementary Fig. 37). The sample temperature under spinning condition was calibrated by KBr reference measurement[60]. In variable temperature ^{1}H MAS NMR measurement, gradual broadening of peak of crystallization water was observed (Supplementary Fig. 39a), as consistent with the results of static ^{1}H NMR. In Supplementary Fig. 39b, Arrhenius plots of FWHM and proton conductivity in the temperature region of 180–270 K for proton conductivity and 196–295 K for MAS NMR are shown. From the proton conductivity, the activation energy of 0.67 eV was obtained. On the other hand, from FWHM, activation energies of 0.16 eV (MAS) was obtained, which would be associated with both rotational and translational motions of water. The difference of activation energies of FWHM and proton conductivity would be derived from difference in observation movement. This value (0.16) is much lower than that of ice (0.59 eV)[61], implying that the molecular motion of confined waters of the present nanotube is active even at low temperature.

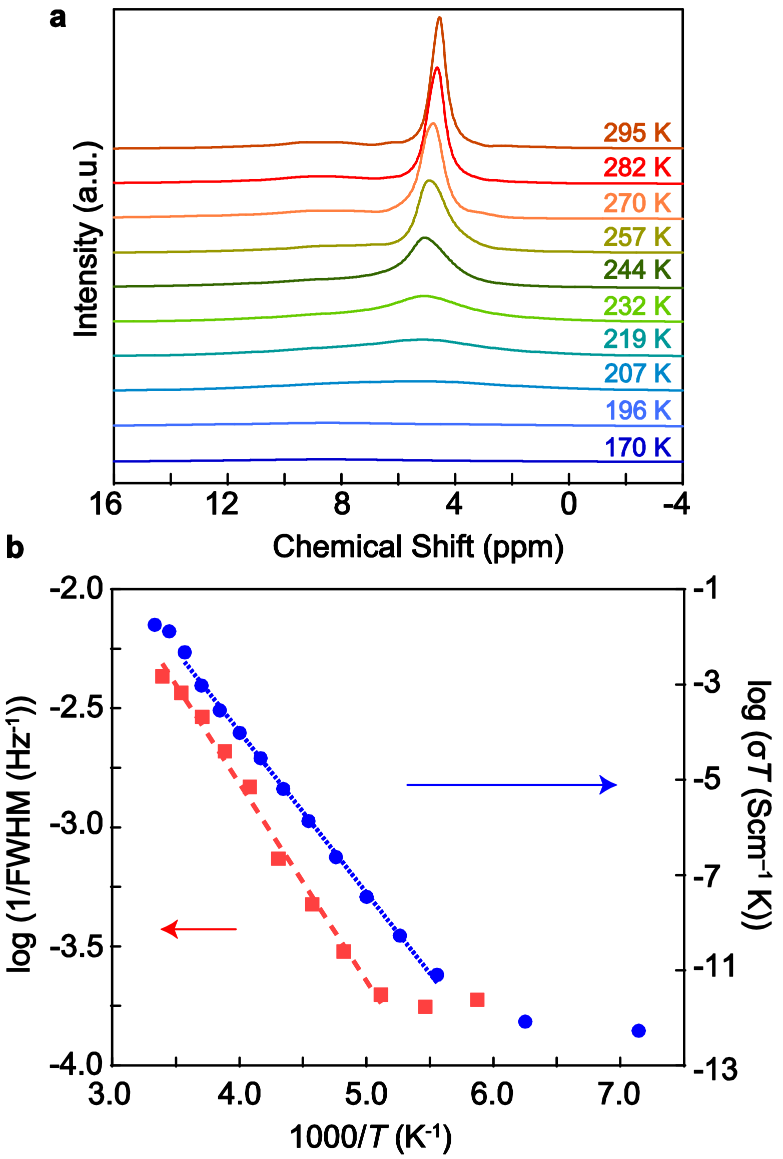

Supplementary Figure 39 | Temperature dependence of full width at half maximum (FWHM) of ^{1}H MAS NMR spectra. a, Temperature dependence of FWHM with decreasing temperature. The measurements were performed with the spin rate of 10 kHz in 400 MHz. **b,** Arrhenius plots of FWHM and proton conductivities. FWHM of ^{1}H MAS NMR results (red square) and proton conductivity (pellet sample: blue circle.) are plotted. The dotted lines indicated the linear fits to the data points.

3. References

(1) Altomare, A. *et al. SIR*92 - a program for automatic solution of crystal structures by direct methods. *J. Appl. Crystallogr.* **27,** 435 (1994).

(2) Beurskens, P. T. *et al*. DIRDIF99: Crystallography Laboratory, University of Nijmegen, The Netherlands, (1999).

(3) Sheldrick, G. M. A short history of *SHELX. Acta Crystallogr., Sect. A* **64,** 112–122 (2008).

(4) CrystalStructure 4.1.0: Crystal Structure Analysis Package, Rigaku and Rigaku/MS.

(5) Nishizawa, H., Nishimura, Y., Kobayashi, M., Irle, S., Nakai, H. Three pillars for achieving quantum mechanical molecular dynamics simulations of huge systems: Divide-and-conquer, density-functional tight-binding, and massively parallel computation. *J. Comput. Chem.* **37**, 1983–1992 (2016).

(6) Martyna, G. J., Klein, M. L., Tuckerman, M. E. Nosé-Hoover chains: The canonical ensemble via continuous dynamics. *J. Chem. Phys.* **97**, 2635–2643 (1992).

(7) Gaus, M., Cui, Q., Elstner, M. DFTB3: Extension of the self-consistent-charge density-functional tight-binding method (SCC-DFTB). *J. Chem. Theory Comput.* **7**, 931–948 (2011).

(8) Gaus, M., Goez, A., Elstner, M. Parametrization and benchmark of DFTB3 for organic molecules. *J. Chem. Theory Comput.* **9**, 338–354 (2013).

(9) Gaus, M., Lu, X., Elstner, M., Cui, Q. Parameterization of DFTB3/3OB for sulfur and phosphorus for chemical and biological applications. *J. Chem. Theory Comput.* **10**, 1518–1537 (2014).

(10) Kubillus, M., Kubař, T., Gaus, M., Řezáč, J., Elstner, M. Parameterization of the DFTB3 method for Br, Ca, Cl, F, I, K, and Na in organic and biological systems. *J. Chem. Theory Comput.* **11**, 332–342 (2015).

(11) Grimme, S., Antony, J., Ehrlich, S., Krieg, H. A consistent and accurate ab initio parametrization of density functional dispersion correction (DFT-D) for the 94 elements H-Pu. *J. Chem. Phys.* **132**, 154104 (2010).

(12) Grimme, S., Ehrlich, S., Goerigk, L. Effect of the Damping Funtion in Dispersion Corrected Density Functional Theory. *J. Comput. Chem.* **32**, 1456–1465 (2011).

(13) Aradi, B., Hourahine, B., Frauenheim, T. DFTB+, a sparse matrix-based implementation of the DFTB method. *J. Phys. Chem. A* **111**, 5678–5684 (2007).

(14) Chou, C.-P., Nishimura, Y., Fan, C.-C., Mazur, G., Irle, S., Witek, H. A. Automatized Parameterization of DFTB Using Particle Swarm Optimization. *J. Chem. Theory Comput.* **12**, 53–64 (2016).

(15) Perdew, J. P., Ruzsinszky, A., Csonka, G. I., Vydrov, O. A., Scuseria, G. E., Constantin, L. A., Zhou, X., Burke, K. Restoring the Density-Gradient Expansion for Exchange in Solids and Surfaces. *Phys. Rev. Lett.* **100**, 136406 (2008).

(16) Blöchl, P. E. Projector augmented-wave method. *Phys. Rev. B* **50**, 17953–17979 (1994).

(17) Kresse, G., Joubert, D. From ultrasoft pseudopotentials to the projector augmented-wave method. *Phys. Rev. B* **59**, 1758–1775 (1999).

(18) Kresse, G., Hafner, J. Ab initio molecular dynamics for liquid metals. *Phys. Rev. B* **47**, 558–561 (1993).

(19) Kresse, G., Hafner, J. Ab initio molecular-dynamics simulation of the liquid-metal–amorphous-semiconductor transition in germanium. *Phys. Rev. B* **49**, 14251–14269 (1994).

(20) Kresse, G., Furthmüller, J. Efficiency of ab-initio total energy calculations for metals and semiconductors using a plane-wave basis set. *Comput. Mater. Sci.* **6**, 15–50 (1996).

(21) Kresse, G., Furthmüller, J. Efficient iterative schemes for *ab initio* total-energy calculations using a plane-wave basis set. *Phys. Rev. B* **54**, 11169–11186 (1996).

(22) Neese, F. The ORCA program system. *Wiley Interdiscip. Rev. Comput. Mol. Sci.* **2**, 73–78 (2012).

(23) Becke, A. D. Density‐functional thermochemistry. III. The role of exact exchange. *J. Chem. Phys.* **98**, 5648–5652 (1993).

(24) Stephens, P. J., Devlin, F. J., Chabalowski, C. F., Frisch, M. J. Ab Initio Calculation of Vibrational Absorption and Circular Dichroism Spectra Using Density Functional Force Fields. *J. Phys. Chem.* **98**, 11623–11627 (1994).

(25) Andrae, D., Häußermann, U., Dolg, M., Stoll, H., Preuß, H. Energy-adjusted ab initio pseudopotentials for the second and third row transition elements. *Theor. Chim. Acta* **77**, 123–141 (1990).

(26) Schäfer, A., Horn, H. &Ahlrichs, R. Fully Optimized Contracted Gaussian-Basis Sets for Atoms Li to Kr. *J. Chem. Phys.* **97**, 2571–2577 (1992).

(27) Fujita, M., Yazaki, J. & Ogura, K. Preparation of a macrocyclic polynuclear complex, $[(en)Pd(4,4'\text{-}bpy)]_4(NO_3)_8$ (en = ethylenediamine, bpy = bipyridine), which recognizes an organic molecule in aqueous media. *J. Am. Chem. Soc.* **112,** 5645–5647 (1990).

(28) Fujita, M., Yazaki, J. & Ogura, K. Spectroscopic Observation of Self-Assembly of a Macrocyclic Tetranuclear Complex Composed of Pt^{2+} and 4,4'-Bipyridine. *Chem. Lett.* 1031–1032 (1991).

(29) Chakrabarty. R., Mukherjee, P. S. & Stang, P. J. Supramolecular Coordination: Self-Assembly of Finite Two- and Three-Dimensional Ensembles. *Chem. Rev.* **111,** 6810–6918 (2011).

(30) Pazout, R. *et al.* A new [(1*R*,2*R*)-1,2-diaminocyclohexane]platinum(II) complex: formation by nitrate-acetonitrile ligand exchange. *Acta Crystallogr., Sect. C* **66,** 273–275 (2010).

(30) Flack, H. D. On enantiomorph-polarity estimation. *Acta Cryst.* **A39,** 876–881 (1983).

(31) Johnstone, T. C. The crystal structure of oxaliplatin: A case of overlooked pseudo symmetry. *Polyhedron* **67,** 429–435 (2014).

(32) Murphy, V. J. *et al.* False Minima and the Perils of a Polar Axis in X-ray Structure Solutions: Molecular Structures of $W(PMe_3)_4H_2X_2$ (X = F, Cl, Br) and $W(PMe_3)_4H_2F_2(H_2O)$. *J. Am. Chem. Soc.* **117,** 9762–9763 (1995).

(33) Yamashita, M., Okamoto, H., Ed., *English, Material Desings and New Physical Properties in MX- and MMX-Chain Compounds* (Springer, Verlag Wien, 2013).

(34) Otsubo, K. *et al.* Bottom-up realization of a porous metal–organic nanotubular assembly. *Nat. Mater.* **10,** 291–295 (2011).

(35) Discovery Studio Visualizer, v.4.5: BIOVIA Inc., (2005–2013).

(36) Hübschle, C. B., Sheldrick, G. M. & Dittrich, B. ShelXle: a Qt graphical user interface for SHELXL. *J. Appl. Crystallogr.* **44,** 1281–1284 (2011).

(37) Bao, S. *et al.* Enhancing Proton Conduction in 2D Co–La Coordination Frameworks by Solid-State Phase Transition. *J. Am. Chem. Soc.* **136,** 9292–9295 (2014).

(38) Sekhaneh, W., Kotecha, M., Dettlaff-Weglikowska, U. & Veeman. W. S. High resolution NMR of water absorbed in single-wall carbon nanotubes. *Chem. Phys. Lett.* **428,** 143–147 (2006).

(39) Morcombe, C. R. & Zilm, K. W. Chemical shift referencing in MAS solid state NMR. *J. Magn. Reson.* **162,** 479–486 (2003).

(40) Stejskal, E. O. & Tanner, J. E. spin diffusion measurements: spin echoes in the presence of a time – dependent field gradient. *J. Chem. Phys.* **42**, 288–292 (1965).

(41) Callaghan, P. T. Translational Dynamics & Magnetic Resonance, OXFORD: New York, 2011.

(42) Stallmach, F. *et al.* Evidence of anisotropic self-diffusion of guest molecules in nanoporous materials of MCM-41, *J. Am. Chem. Soc.* **122,** 9237–9242 (2000).

(43) Callaghan, P. T. & Jolley, K. W. Diffusion of water in the endosperm tissue of wheat grains as studied by pulsed field gradient nuclear magnetic resonance. *Biophys. J.* **28,** 133–141 (1979).

(44) Qiao, Y., Galvosas, P. & Callaghan, P. T. Diffusion correlation NMR spectroscopic study of anisotropic diffusion of water in plant tissues. *Biophy. J.*, **89,** 2899–2905 (2005).

(45) Zhurko, G. A. Chemcraft. http://www.chemcraftprog.com

(46) Granovsky, A. A., Firefly version 8, http://classic.chem.msu.su/gran/firefly/index.html

(47) Schmidt, M. W. *et al.* General atomic and molecular electronic structure system. *J. Comput. Chem.* **14,** 1347–1363 (1993).

(48) http://research.chem.psu.edu/brpgroup/pKa_compilation.pdf, and the references therein.

(49) Elstner, M. *et al*. Self-consistent-charge density-functional tight-binding method for simulations of complex materials properties. *Phys. Rev. B* **58**, 7260–7268 (1998).

(50) Perdew, J. P., Burke, K. & Ernzerhof, M. Generalized Gradient Approximation Made Simple. *Phys. Rev. Lett.* **77**, 3865–3868 (1996).

(51) van Lenthe, E. Ehlers, A. & Baerends, E.-J. Geometry optimizations in the zero order regular approximation for relativistic effects. *J. Chem. Phys.* **110**, 8943–8953 (1999).

(52) Gaus, M., Chou, C.-P., Witek, H. & Elstner, M. Automatized parametrization of SCC-DFTB repulsive potentials: application to hydrocarbons. *J. Phys. Chem. A* **113**, 11866–11881 (2009).

(53) Maupin, C. M., Aradi, B., Voth, G. A. The self-consistent charge density functional tight binding method applied to liquid water and the hydrated excess proton: Benchmark simulations. *J. Phys. Chem. B.* **114**, 6922–6931 (2010).

(54) Nakai, H., Sakti, A. W., Nishimura, Y., Divide-and-Conquer-Type Density-Functional Tight-Binding Molecular Dynamics Simulations of Proton Diffusion in a Bulk Water System, *J. Phys. Chem. B.* **120**, 217−221 (2016).

(55) Sakti, A. W., Nishimura, Y. & Nakai, H. Divide-and-Conquer-Type Density-Functional Tight-Binding Simulations of Hydroxide Ion Diffusion in Bulk Water. *J. Phys. Chem. B* **121**, 1362–1371 (2017).

(56) Markovitch, O. *et al.* Special Pair Dance and Partner Selection: Elementary Steps in Proton Transport in Liquid Water. *J. Phys. Chem. B* **112**, 9456–9466 (2008).

(57) Mills, R., Self-diffusion in normal and heavy water in the range 1-45.deg. *J. Phys. Chem.* **77**, 685–688 (1973).

(58) Meiboom, S. Nuclear magnetic resonance study of the proton transfer in water. *J. Chem. Phys.*34, 375–388 (1961).

(59) Roberts, N. K., Northey, H. L. Proton and deuteron mobility in normal and heavy water solutions of electrolytes. *J. Chem. Soc. Faraday Trans*. **70**, 253–262 (1974).

(60) Thurber, K. R. & Tycko, R. Measurement of sample temperatures under magic-angle spinning from the chemical shift and spin-lattice relaxation rate of ^{79}Br in KBr powder. *J. Magn. Res.* **196,** 84–87 (2009).

(61) Wittebort, R. J., Usha, M. G., Ruben, D. J., Wemmer, D. E. & Pines, A. Observation of molecular reorientation in ice by proton and deuterium magnetic resonance. *J. Am. Chem. Soc.* **110,** 5668–5671 (1988).
