# Supplementary material for: Confined water-mediated high proton conduction in hydrophobic channel of a synthetic nanotube
Source: Nat Commun. 2020 Feb 18;11:843. doi: 10.1038/s41467-020-14627-z (PMC7029035; doi:10.1038/s41467-020-14627-z)
Supplement: Supplementary file 2 — Description of Additional Supplementary Files [file 41467_2020_14627_MOESM2_ESM.docx]

**Description of Additional Supplementary Files**

File Name: Supplementary Data 1

Description: This file is crystallographic information for compound **1** in cif format.

File Name: Supplementary Data 2

Description: This file is crystallographic information for Pt(II) square complex in cif format.

File Name: Supplementary Data 3

Description: This file is crystallographic information for Pt(IV) square complex in cif format.

File Name: Supplementary Movie 1

Description: The trajectory of hydronium 1 (within channel A) from 0 to 1.5 ps in orthographic view.

File Name: Supplementary Movie 2

Description: The trajectory that the hydronium 1 migrates from channel A to channel B from 3.0 to 6.33 ps. The movie during the migration (around 4.2 ps) has been created using slow motion (10 times of the number of frames, or 0.1×) for clarity.

File Name: Supplementary Movie 3

Description: The trajectory of hydronium 2 (within channel B) from 7.5 to 9 ps in orthographic view.
